# Data-Driven Development of Biomedical Hydrogels for Controlled Drug Delivery: Clinical Applications and Emerging Machine-Learning Approaches

**DOI:** 10.3390/jfb17090444

**Published:** 2026-09-02

**Authors:** Elham Eskandarnia, Ayah Binrajab, Adnan Alsaei, Fatema Rahimi, Nasser Alahmed, Ahmad Zarwi, G. Roshan Deen

**Affiliations:** 1 Faculty of Computer Studies, Arab Open University, Building 890, Street 3220, A’ali 732, Bahrain; 2 Materials for Medicine Research Group, School of Medicine, Royal College of Surgeons in Ireland (RCSI), Medical University of Bahrain, Busaiteen 228, Bahrain; 24200305@rcsi.com (A.B.); 23200122@rcsi.com (F.R.); 23201339@rcsi.com (N.A.); 24204621@rcsi.com (A.Z.); rdeen@rcsi.com (G.R.D.)

**Keywords:** biomedical hydrogels, controlled drug delivery, hydrogel synthesis, formulation optimisation, machine learning, stimuli-responsive hydrogels

## Abstract

Hydrogels are hydrated three-dimensional polymeric networks with biomedical potential because they can encapsulate therapeutic agents and provide localised, sustained, or stimulus-responsive drug delivery. Their performance is determined by interacting variables, including polymer composition, synthesis route, crosslinking chemistry, drug loading, swelling, degradation, and the biological microenvironment. This multidimensional design space often makes hydrogel development slow and dependent on trial-and-error experimentation. This review examines the data-driven development of biomedical hydrogels for controlled drug delivery, focusing on clinical applications and emerging machine-learning approaches that support material selection, formulation design, synthesis optimisation, and release prediction. The review first discusses natural and synthetic hydrogels, including alginate, chitosan, gelatin-based systems, hyaluronic acid, and polyethylene glycol, with emphasis on how their physicochemical properties influence biocompatibility, synthesis flexibility, and drug-release behaviour. Key applications are then considered, including wound healing, cancer therapy, glucose-responsive insulin delivery, and inflammatory disease management. Particular attention is given to injectable and stimuli-responsive hydrogels, where formulation conditions and synthesis parameters can be tuned to improve localisation, therapeutic exposure, and release control. The review evaluates machine-learning methods, including random forest, gradient boosting, artificial neural networks, Gaussian process regression, and active learning, for predicting hydrogel properties, modelling release profiles, optimizing synthesis and formulation variables, and prioritizing experimental candidates. Finally, translational challenges are addressed, including small non-standardised datasets, limited external validation, weak in vitro-clinical correlations, material safety, explainability, reproducibility, scalability, and regulatory requirements. By integrating clinical, materials, synthesis, and machine-learning perspectives, this review highlights opportunities for developing safer and clinically relevant hydrogel-based drug-delivery systems.

## 1. Introduction

Effective pharmacotherapy depends not only on selecting an active therapeutic agent but also on delivering it to the appropriate site at a suitable concentration and for a sufficient duration. Conventional systemic administration can produce fluctuating drug concentrations, expose healthy tissues to pharmacological activity, and require repeated dosing, particularly when an agent has a short biological half-life or limited stability. Controlled drug-delivery systems are intended to address these limitations by regulating the spatial and temporal availability of therapeutics. In principle, maintaining drug concentrations within the therapeutic range and concentrating exposure at the intended site can improve efficacy, reduce off-target toxicity and decrease the frequency of administration [1]. Localised delivery is therefore especially relevant when treatment is required within a defined tissue or pathological environment, including wounds, solid tumours, and inflamed or damaged musculoskeletal tissues [1,2].

Hydrogels have emerged as promising platforms for achieving this control. They are three-dimensional networks of natural, synthetic, or semi-synthetic polymers that are crosslinked physically or chemically and can retain large quantities of water or biological fluid [2]. Their hydrated structure resembles several features of soft biological tissues, while their composition, architecture, and mechanical behaviour can be adjusted for different routes of administration and therapeutic requirements. Hydrogels may encapsulate a broad range of agents, including small-molecule drugs, proteins, nucleic acids, and cells, and their aqueous preparation conditions can help protect sensitive therapeutics from denaturation or premature degradation [1,2]. Depending on their design, they may be implanted, applied topically, or delivered through minimally invasive injection, allowing a therapeutic depot to be formed close to the target site. These characteristics have supported investigation of hydrogel-based delivery in wound management, cancer treatment, diabetes, inflammatory disease, and tissue repair [2,3].

The effectiveness of a hydrogel delivery system is determined by its formulation as well as by the intrinsic properties of the incorporated drug. Polymer identity and concentration, molecular weight, crosslinking method and density, solvent conditions, porosity, mesh size, and degradation mechanism can influence gelation, injectability, swelling, mechanical stability, biocompatibility, and residence time. At the same time, drug size, charge, solubility, and affinity for the polymer network affect loading efficiency, diffusion, and release kinetics [1]. Drug release may occur through diffusion, network swelling, matrix degradation, cleavage of a drug–polymer linkage or a combination of these mechanisms. Stimuli-responsive hydrogels add a further level of control by altering their structure or interactions in response to changes in temperature, pH, glucose concentration, enzyme activity, or other features of the local environment [1,3]. Consequently, hydrogel formulation and drug-delivery performance should not be treated as separate design problems: formulation choices establish the physical and chemical conditions that ultimately govern therapeutic loading, retention, and release.

Despite this versatility, developing a hydrogel that simultaneously satisfies material, biological, and pharmaceutical requirements remains difficult. The formulation space is multidimensional, and changing one component can affect several outcomes in different or competing ways. For example, increasing crosslinking density may improve mechanical stability but reduce swelling, alter degradation, and restrict drug diffusion. Conventional development therefore commonly relies on sequential experimentation and trial-and-error adjustment of individual variables. This approach can be expensive and time consuming, may explore only a small part of the available design space, and can overlook nonlinear interactions among formulation parameters [4]. Translation is further complicated by differences in material characterisation, release-testing conditions, and biological models across studies. A lack of standardised methods limits comparison between formulations, reduces reproducibility, and weakens the connection between in vitro release measurements and in vivo performance [2].

Machine learning (ML) offers a complementary, data-driven approach to these challenges. Rather than evaluating formulation variables independently, supervised learning algorithms can model relationships between multiple inputs, such as polymer composition, component concentrations, crosslinking conditions, and drug characteristics and outputs such as gel formation, swelling, degradation, mechanical properties, loading efficiency, and release profiles [4,5]. Classification models can help identify formulations likely to form stable hydrogels, while regression models can predict continuous performance measures. Feature-importance and explainability methods may also reveal which variables most strongly influence a target property. When combined with Bayesian optimisation or active learning, ML can prioritise the next experiments expected to provide the greatest improvement or information, thereby supporting more efficient exploration of complex formulation spaces [5,6]. Recent studies have demonstrated the use of ML to optimise several hydrogel properties simultaneously and to predict the hydrogel-forming ability of candidate molecules, illustrating its potential to guide both formulation selection and experimental validation [6,7].

However, ML should be regarded as a tool for supporting, rather than replacing, experimental and clinical judgement. Model performance depends on the quality, size, and representativeness of the training data, yet hydrogel datasets are often small, heterogeneous, and generated using non-standardised protocols. Predictions may not generalise to new materials, drugs, or biological settings without external validation, and technically accurate models may still have limited translational value if their outcomes are not linked to biocompatibility, therapeutic efficacy, and clinically relevant release behaviour [2,5]. Explainability, prospective experimental testing and careful reporting of negative as well as positive results are therefore essential to the responsible application of ML in this field.

Accordingly, this review examines biomedical hydrogels through two connected perspectives: the formulation of hydrogel materials with suitable physicochemical and biological properties, and the control of drug loading and release from those systems. It first considers clinically relevant natural and synthetic hydrogel materials and the properties that determine their performance. It then discusses controlled-delivery mechanisms, injectable and stimuli-responsive strategies, and representative applications in wound healing, local cancer therapy, glucose-responsive insulin delivery, and musculoskeletal or inflammatory conditions. Finally, it evaluates emerging ML approaches for predicting hydrogel behaviour, optimising formulation variables, and selecting informative experiments, while critically considering the data, validation, safety, explainability, and regulatory challenges that must be addressed for clinical translation.

## 2. Biomedical Hydrogels: Materials and Relevant Properties

The clinical performance of any hydrogel-based drug-delivery system is dictated, first and foremost, by the chemistry of its constituent polymer network [8]. Whether derived from natural biopolymers or synthesised de novo, the material selected determines the crosslinking chemistry available, the resulting mesh architecture, and ultimately the manner in which an incorporated therapeutic agent is retained and released [9]. Li and Mooney frame this as a problem that plays out across several length scales at once: a macroscopic scale, where gel size and porosity dictate whether a hydrogel can be implanted, injected or applied topically; a mesh scale, spanning only a few nanometres, that governs how freely a drug can diffuse through the network; and a molecular scale, at which specific chemical interactions between drug and polymer take place [8]. Because these scales can be engineered largely independently, the same base chemistry can be steered toward very different release profiles by adjusting concentration, crosslink density, or the presence of specific binding motifs. This section reviews the principal natural and synthetic hydrogel materials used in biomedical practice then examines the physicochemical properties that govern clinical suitability, namely biocompatibility, biodegradability, swelling, and mechanical stability, and how these properties interact with drug loading and release.

### 2.1. Natural Hydrogels: Alginate, Chitosan, Gelatin/GelMA, and Hyaluronic Acid

Natural, biopolymer-derived hydrogels remain attractive drug carriers because their chemical resemblance to native extracellular matrix components confers intrinsic biocompatibility and, in most cases, enzymatic biodegradability [9]. Alginate, a linear anionic polysaccharide extracted from brown algae, gels almost instantaneously in the presence of divalent cations such as Ca^2+^, which bridge adjacent guluronic acid blocks in an “egg-box” ionic crosslink. This mild, aqueous gelation makes alginate one of the most widely used matrices for encapsulating labile biologics and cells, although its ionotropic network is prone to uncontrolled ion-exchange dissolution under physiological conditions, which can compromise long-term release control [10].

Chitosan, a cationic polysaccharide derived from chitin deacetylation, complements alginate through electrostatic interaction and additionally offers mucoadhesive and antimicrobial properties useful for wound and mucosal delivery. Because chitosan is only readily soluble, and therefore only gels, under mildly acidic conditions, it is frequently combined with alginate or other anionic polymers, or chemically modified, to form stable networks at physiological pH [10].

Gelatin, a denatured derivative of collagen, retains cell-adhesive arginine-glycine-aspartic acid (RGD) motifs and protease-cleavable peptide sequences from its parent protein, but its thermoreversibility limits its use as an unmodified hydrogel [11]. Reacting gelatin with methacrylic anhydride grafts methacryloyl groups onto the free amine and hydroxyl residues along the protein backbone, giving gelatin methacryloyl (GelMA), which crosslinks permanently under ultraviolet light once a water-soluble photoinitiator such as Irgacure 2959 or lithium acylphosphinate is added. The degree of methacryloyl substitution (DS), controlled simply by varying the amount of methacrylic anhydride and the reaction pH, has become the main lever for tuning GelMA properties: raising DS from roughly 50% to 73% has been shown to increase compressive modulus from about 2 kPa to 4.5 kPa while the average pore size falls from around 50 μm to 25 μm, and higher GelMA concentration produces a comparable stiffening effect. Because methacrylation reacts with only a small fraction of gelatin’s amino acid residues and leaves the RGD sequence untouched, cell adhesion and matrix metalloproteinase (MMP)-mediated degradability are largely preserved, which explains why GelMA has become a default choice wherever a photocrosslinkable, cell-compatible carrier is needed. GelMA systems have since been used to deliver antibiotics, growth factors, and small-molecule chemotherapeutics, with encapsulation efficiencies exceeding 90% in some formulations [11].

Hyaluronic acid (HA), a glycosaminoglycan ubiquitous in connective and synovial tissue, is valued for its viscoelasticity and receptor-mediated interaction with cell-surface CD44, which can be exploited for tumour-targeted delivery. Because unmodified HA has limited structural stability and durability, crosslinking is commonly used to produce more stable HA-based hydrogels. In current clinical applications, particularly HA-based dermal fillers, chemical crosslinking is widely employed to improve material stability and prolong persistence after administration [12].

A group of natural hydrogel biomaterials are used for drug delivery and applications. The major subtypes are summarised in Table 1.

### 2.2. Synthetic Hydrogels: Polyethylene Glycol, Polyvinyl Alcohol, and Related Systems

Where natural polymers offer bioactivity at the cost of batch-to-batch variability, fully synthetic hydrogels prioritise reproducibility and tunability. Poloxamers (Pluronics), amphiphilic PEO-PPO-PEO triblock copolymers, represent an important class of synthetic polymers with pharmaceutical and biomedical applications, particularly because selected grades exhibit thermoreversible behaviour [13]. Polyethylene glycol (PEG) is the most extensively studied synthetic hydrogel material in drug delivery, owing to its hydrophilicity, negligible immunogenicity, and the ease with which its terminal hydroxyl groups can be functionalised with acrylate, thiol, or other reactive handles to permit controlled photopolymerisation. The resulting networks are essentially bio-inert, allowing release kinetics to be governed largely by mesh size and diffusion rather than by specific polymer–drug interactions. This same inertness, however, limits cell adhesion and can necessitate co-formulation with bioactive motifs for tissue-contacting uses [14].

Polyvinyl alcohol (PVA) forms hydrogels through a distinct, additive-free mechanism: repeated freeze–thaw cycling induces crystalline domains that physically crosslink the polymer chains, yielding tough, elastic networks without chemical initiators or crosslinkers. This is attractive for load-bearing applications such as cartilage substitutes, though physically crosslinked PVA gels can be prone to slow dissolution or syneresis in aqueous physiological environments over extended periods. Blending PVA with PEG, or with polyacrylic acid, is a common strategy to stabilise the hydrogen-bonded network, tune swelling, and provide an additional handle for modulating drug-release rate [15]. Between them, natural and synthetic chemistries cover a stiffness range of roughly three orders of magnitude, from about 0.5 kPa up to 5 MPa, so a network can in principle be formulated to approximate the mechanical properties of almost any soft tissue [8].

Poloxamers (Pluronics) are another important class of synthetic polymers used in hydrogel-based drug delivery. They are amphiphilic triblock copolymers composed of poly(ethylene oxide)-poly(propylene oxide)-poly(ethylene oxide) (PEO-PPO-PEO), with different grades exhibiting distinct physicochemical properties depending on their molecular weight and PEO/PPO composition. A particularly important characteristic of selected poloxamers is their thermoreversible behaviour, which enables temperature-dependent changes in their physical state. This property makes poloxamer-based formulations particularly attractive for pharmaceutical and biomedical applications, including drug-delivery systems. Differences among poloxamer grades can substantially influence their physicochemical and functional properties and should therefore be considered when selecting a poloxamer for a specific therapeutic application [13].

The main synthetic hydrogels used in drug delivery and pharmaceutical devices are summarised in Table 2.

### 2.3. Clinically Relevant Properties: Biocompatibility, Biodegradability, Swelling, and Mechanical Stability

Irrespective of origin, four interrelated properties determine whether a hydrogel is fit for clinical drug delivery. Biocompatibility describes the absence of an adverse local or systemic response and depends on both the parent polymer and any residual crosslinking agents or initiators; natural polymers generally perform favourably here, whereas synthetic networks require careful removal of unreacted monomer and photoinitiator by-products [9]. Biodegradability, whether hydrolytic (as in ester-linked PEG derivatives) or enzymatic (as in protease-cleavable GelMA), determines whether a depot must be surgically retrieved or is safely cleared, and dictates the duration over which the drug reservoir persists in vivo [11]. Swelling behaviour reflects the balance between the network’s elastic retraction and its osmotic affinity for water, and is most commonly quantified via equilibrium swelling ratio; because swelling expands the hydrogel mesh, it directly influences both solute diffusion rate and the network’s accessibility to enzymatic or hydrolytic attack. Mechanical stability, typically characterised by compressive or storage modulus, must be matched to the implantation site, since excessively soft networks may fail under physiological loading or injection forces, while excessively stiff networks compromise conformability and patient comfort. This mechanical response is not purely elastic, either: networks held together by strong covalent crosslinks behave essentially as elastic solids, whereas those stabilised by weaker secondary bonds, such as the hydrogen bonding in agarose or the hydrophobic association in hydroxypropyl methylcellulose, dissipate stress by continually breaking and reforming crosslinks and are therefore viscoelastic; the timescale over which this occurs, the relaxation time, adds a design variable that is largely independent of stiffness itself. Notably, these properties are not independent: increasing crosslink density to raise stiffness invariably reduces both swelling capacity and mesh size, illustrating the design trade-offs inherent to hydrogel formulation [16].

**Figure 1:**
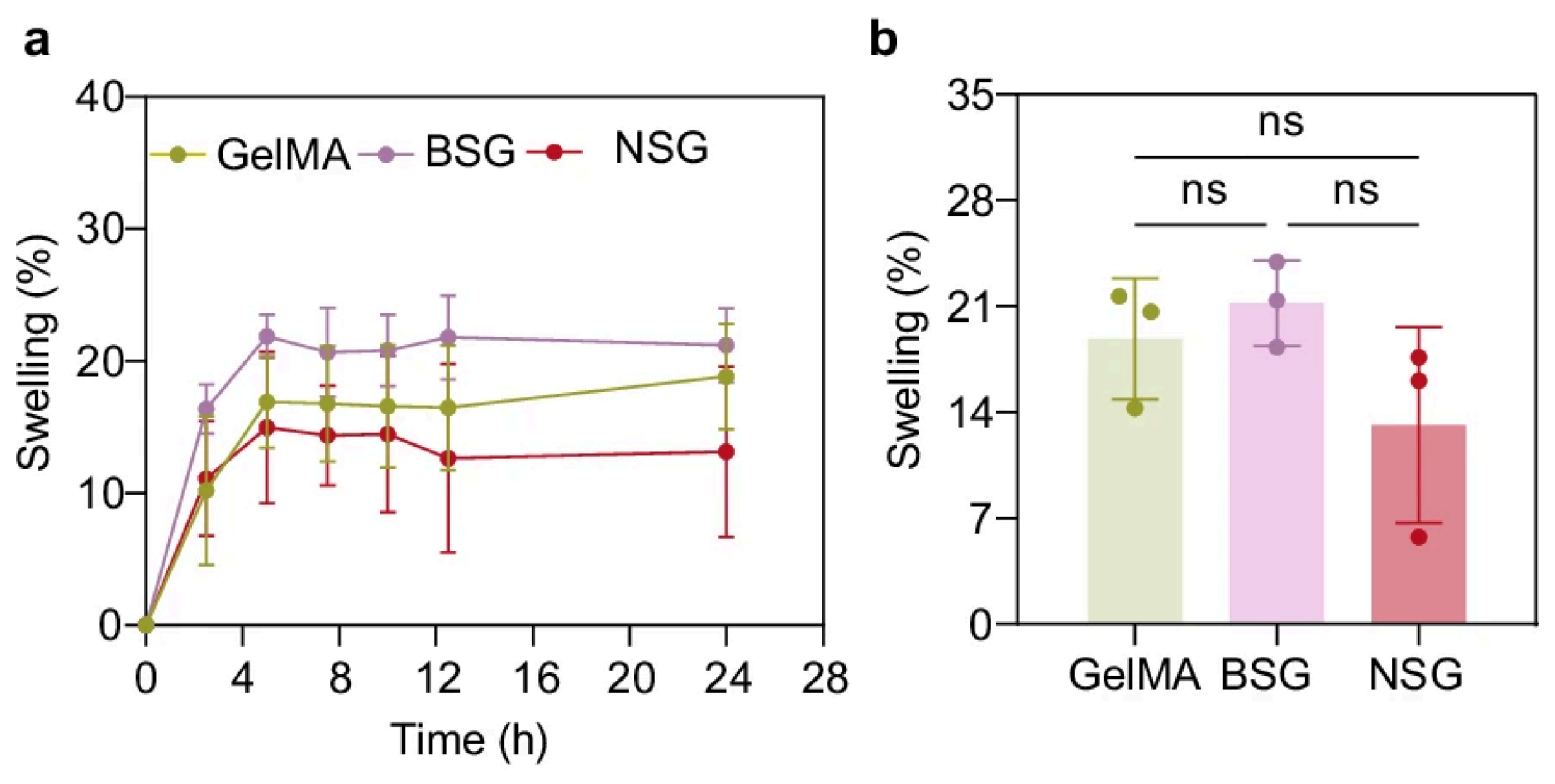
Swelling characteristics of GelMA, bundle-supported GelMA (BSG), and nanorod-supported GelMA (NSG) hydrogels in phosphate-buffered saline. (**a**) Time-dependent swelling over 24 h. (**b**) Swelling measured at 24 h, showing no significant differences among the groups. ns, not significant. Reprinted from Ref. [17].

A recent example illustrates how reinforcement strategies interact with these swelling and degradation properties in practice. Zhu et al. compared unmodified GelMA with two peptide-nanorod-reinforced variants, bundle-supported GelMA (BSG) and nanorod-supported GelMA (NSG), both of which incorporate short, chemically grafted peptide rods to toughen the network. All three hydrogels reached equilibrium swelling within 24 h in phosphate-buffered saline, while quantitative comparison at 24 h confirmed that the swelling values did not differ significantly among the groups (Figure 1a,b), indicating that grafting rigid peptide nanostructures onto the GelMA backbone does not substantially alter water uptake or, by extension, the mesh dimensions available for solute diffusion [17].

**Figure 2:**
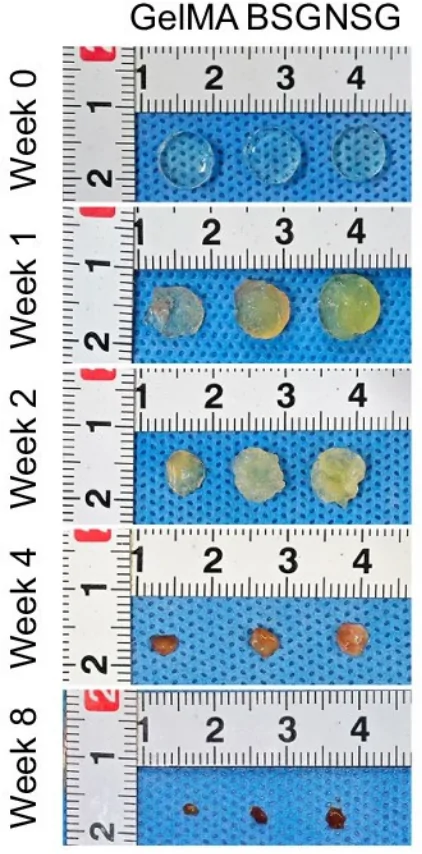
Macroscopic degradation of GelMA, bundle-supported GelMA (BSG), and nanorod-supported GelMA (NSG) hydrogel discs retrieved from the subcutaneous dorsal tissue of rats at 0, 1, 2, 4, and 8 weeks after implantation. Reprinted from Ref. [17].

Subcutaneous implantation in rats told a different story for degradation: photographs of retrieved discs taken at 0, 1, 2, 4, and 8 weeks show that BSG and NSG persisted visibly longer than plain GelMA, which had already fragmented into small opaque remnants by week 4 (Figure 2) [17]. The authors attribute this slower clearance to the denser, smaller-pore network created by the peptide crosslinks, consistent with the general principle that raising crosslink density reduces mesh size and, with it, the rate of hydrolytic or enzymatic erosion [16,17].

The example is a useful illustration that swelling capacity and biodegradation rate, while both governed by network architecture, are not always coupled in the same direction, since a reinforcement strategy can leave one largely unchanged while substantially altering the other.

The material characteristics discussed in this section represent potential input variables for data-driven hydrogel development. Polymer type, concentration, molecular weight, crosslinking mechanism, swelling, degradation, and mechanical modulus can be incorporated as model features for predicting formulation performance and selecting candidates for experimental evaluation.

### 2.4. Influence of Hydrogel Composition on Drug-Loading and -Release Behaviour

The relationship between formulation variables and release outcomes can be described, for diffusion-dominated systems, through classical polymer network theory. Hydrogel mesh sizes typically fall between about 5 and 100 nm, a range comparable to the hydrodynamic diameter of many protein drugs; so whether a molecule diffuses out quickly, slowly, or barely at all depends largely on how the mesh size compares with the size of the drug itself. When the mesh is markedly larger than the drug, diffusion is close to unimpeded and follows simple Stokes–Einstein kinetics; as the two converge, steric drag slows transport and extends release; and once the drug exceeds the mesh, it stays trapped until the network degrades or swells [8]. The Flory–Rehner framework relates equilibrium swelling to crosslink density and, from this, allows estimation of the average mesh size, the correlation length between adjacent crosslinks, which represents the principal steric barrier a diffusing drug molecule must negotiate [16]. Increasing polymer concentration or crosslinker ratio reduces mesh size and slows release, whereas macromolecular therapeutics larger than the mesh become effectively entrapped until the network itself degrades, shifting the release mechanism from diffusion-controlled to degradation-controlled [16]. Drug physicochemical characteristics compound these network effects: hydrophobic or polymer-affinitive drugs partition preferentially into the network and are released more slowly than hydrophilic solutes of comparable size, and ionic drugs can interact electrostatically with charged natural polymers such as alginate or chitosan, producing additional retardation or, conversely, competitive-exchange-triggered burst release [10]. For physically crosslinked or degradable gels, a further consideration is the relative speed of solvent diffusion against network relaxation: comparing the diffusion time with the relaxation time indicates whether release will follow ordinary Fickian kinetics or a slower, non-Fickian pattern tied to a moving glassy-to-rubbery front within the gel [16].

## 3. Hydrogel-Based Strategies for Controlled Drug Delivery

Hydrogels are new drug-delivery systems for the controlled and sustained release of therapeutic agents by diffusion, swelling, or degradation of the polymeric matrices. Injectable and stimuli-responsive hydrogels such as pH-, temperature-, glucose-, and enzyme- sensitive systems offer the possibility of targeted and on-demand medication delivery. However, the challenges for wider clinical use include insufficient mechanical strength, rapid release, and scalability, in addition to their remarkable biocompatibility and adaptability.

### 3.1. Mechanisms of Drug Encapsulation and Release

Therapeutic agents can either be added to a hydrogel while the network is forming by mixing them into the precursor solution prior to crosslinking, or after the gel has been made by allowing the drug to diffuse from an external source into the pre-formed hydrogel. In this second method, the drug interacts with functional groups of the polymer over time such as hydroxyl groups which aids in its incorporation into the network. The method of incorporation and the resulting drug–polymer interactions will ultimately determine how a drug will behave once released from a hydrogel [18].

**Figure 3:**
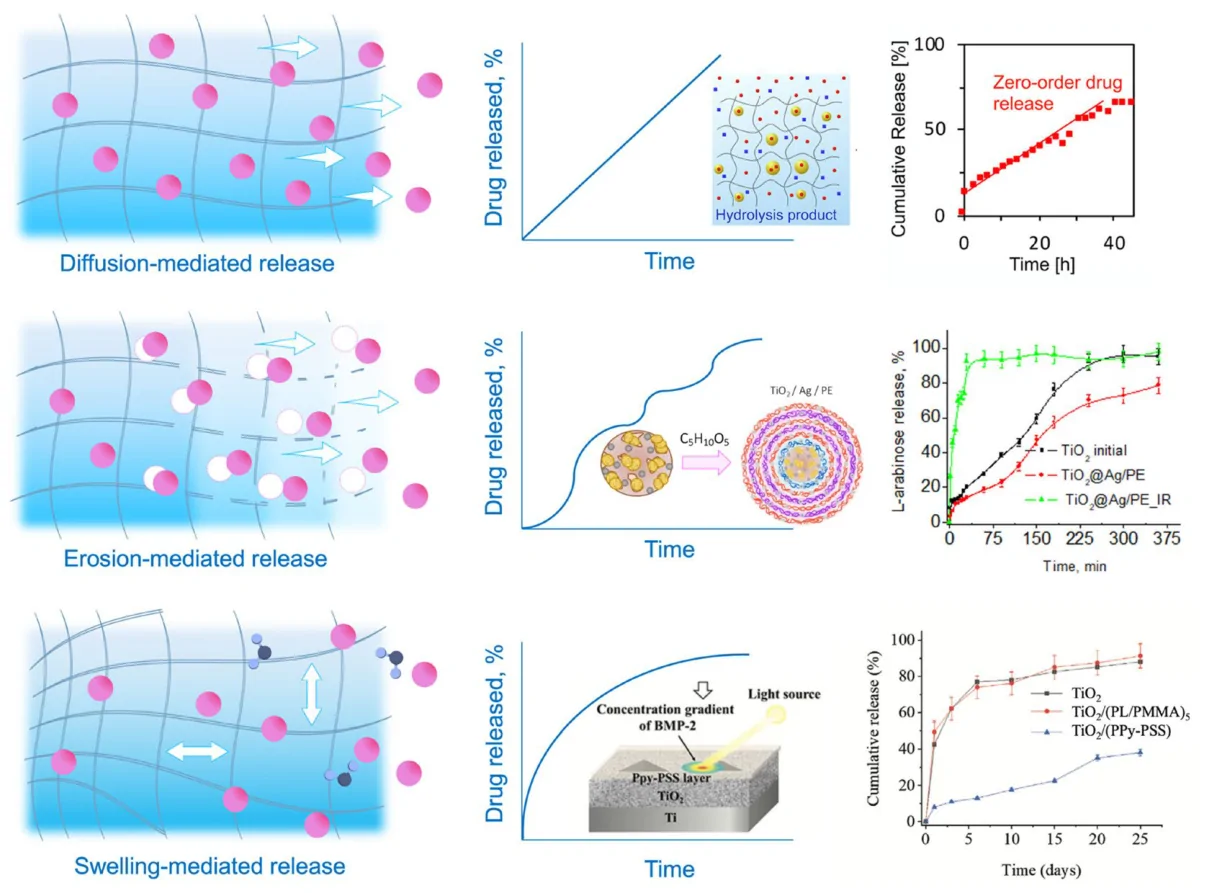
Major mechanisms of drug release from hydrogel-based delivery systems. Reprinted from Ref. [21].

Upon loading, drugs can be released from the hydrogel network via diffusion, swelling, degradation, and external stimuli such as temperature and pH as shown in Figure 3. Depending upon the polymer composition and the network structure, these mechanisms can be engineered to achieve controlled or sustained drug release from the system [19,20]. In diffusion-controlled systems, drug molecules move out from a region of high concentration inside the gel matrix to the adjacent biological fluid along a concentration gradient and the hydrogel acts as a reservoir. This is generally regarded as the most important release mechanism. The rate of this release mechanism is mainly controlled by the physical interaction of the drug with the surrounding polymer matrix [21].

**Figure 4:**
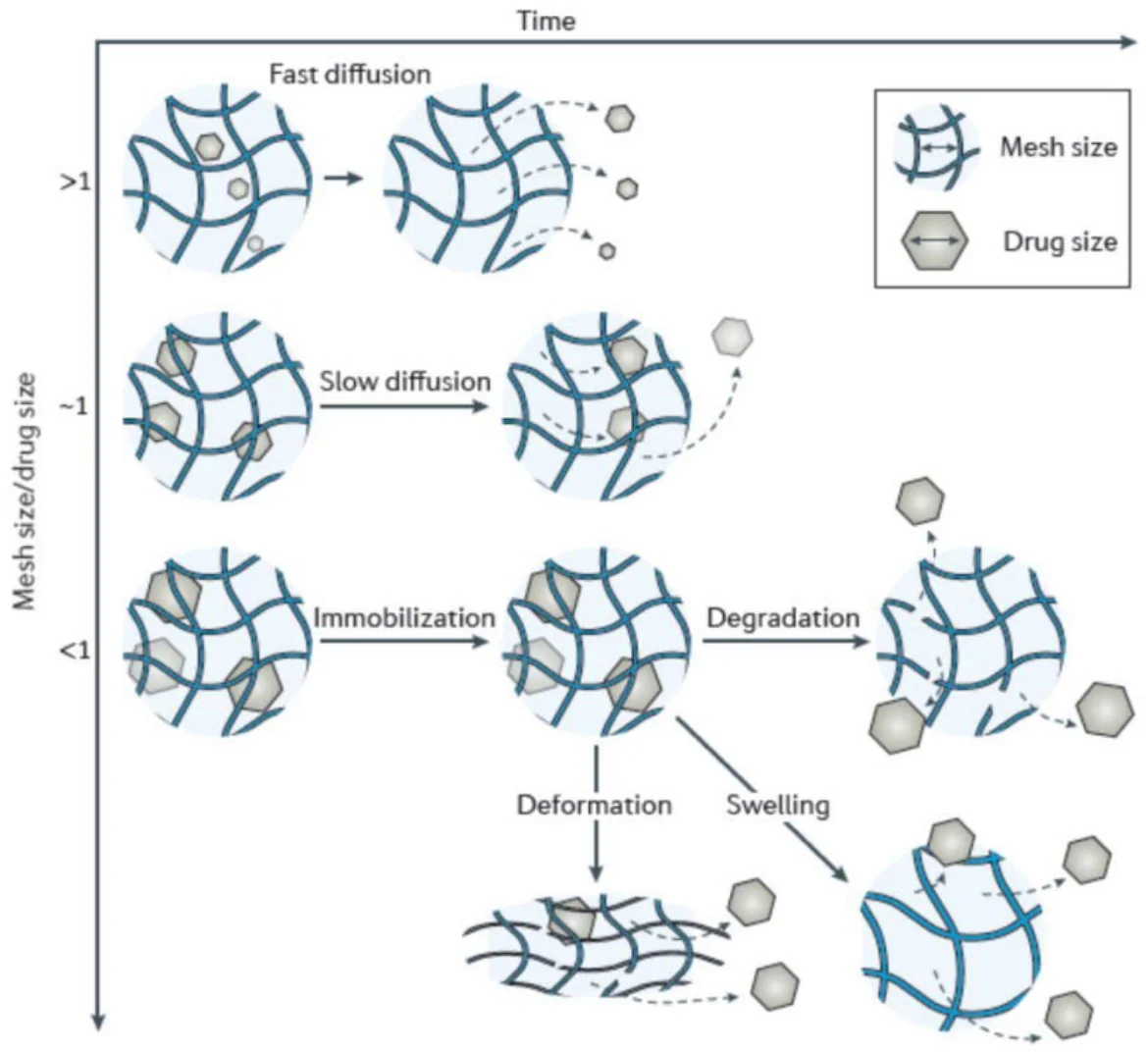
Influence of hydrogel mesh size relative to drug size on drug-release mechanisms. Reprinted from Ref. [22].

This relation is best described in terms of the mesh size, which is defined as the mean distance between neighbouring polymer chains in the crosslinked network. When the drug is much smaller than the mesh size, diffusion proceeds at a rapid rate, when the drug is similar in size to the mesh size, diffusion can be dramatically slowed, and when the drug is larger than the mesh size, diffusion is essentially blocked and the drug must be released through degradation, swelling, or deformation of the network as shown in Figure 4 [19,22]. This size-dependent behaviour accounts for the large variation in release profiles of different therapeutic molecules for the same hydrogel formulation and, further, why the network architecture needs to be purposely tuned based on the molecular size of the therapeutic agent of interest [23].

Swelling-controlled release becomes particularly relevant when a hydrogel is initially in a glassy or low-hydration state. In these systems, the drug is trapped within a glassy gel matrix, and upon contact with biological fluids, the gel swells beyond its original boundary; this swelling causes relaxation of the polymer chains, which permits drug diffusion outward in a process referred to as anomalous transport, since it does not follow simple Fickian diffusion kinetics [24]. The rate-limiting step in such systems is therefore not the drug’s intrinsic diffusivity but the rate at which the polymer network itself reorganises in response to water uptake [25].

A third important mechanism is degradation-controlled release, whereby the drug is largely immobilised within the network until the surrounding polymer matrix degrades. The release of drugs in biodegradable hydrogels is through polymer chain cleavage and matrix erosion [26,27]. During hydrolysis, enzymatic activity, or other mechanisms, the hydrogel degrades and the encapsulated drug is released in a controlled manner [22]. This mechanism is particularly advantageous where sustained release over extended periods is desired, as the release kinetics can be manipulated by altering the chemistry and density of degradable linkages within the network, rather than solely relying on diffusion-driven transport [28]. The chemical parameters overlap but are not identical, which allows formulation engineering to decouple the degradation and drug-release rates, permitting slow degradation of a hydrogel with drug release at a clinically relevant rate [29]. The three principal hydrogel drug-release mechanisms differ in their driving forces, rate-controlling factors, advantages, and limitations, as summarised in Table 3.

### 3.2. Injectable Hydrogel

Historically, hydrogel scaffolds have been made externally and delivered via invasive surgery. Advances in polymer chemistry and materials science have enabled the formation of hydrogel networks in situ after minimally invasive delivery by a standard syringe or needle [30,31]. This approach could greatly diminish or even eliminate the need for surgical insertion. A typical in situ gelling system is the direct injection of a liquid precursor solution containing the therapeutic agent into the target tissue where it gels under physiological conditions. This localised gel formation allows for better spatial and temporal control over the drug delivery, which may increase therapeutic efficacy and reduce systemic toxicity and off-target effects as well as the overall drug dosage [32].

An injectable hydrogel can be defined as a hydrated three-dimensional polymer network that is administered through a syringe or catheter, either as a free-flowing precursor that subsequently gels in situ or as a preformed shear-thinning material that temporarily flows under applied force. Its suitability for injection depends on interrelated physicochemical properties, including viscosity, shear-thinning behaviour, injection force, gelation time, crosslinking mechanism, swelling, degradation, mesh size, mechanical strength, and drug diffusivity. These properties determine its ability to pass through the needle, conform to the target tissue, remain stable after administration, and provide the required drug-release profile [33].

**Figure 5:**
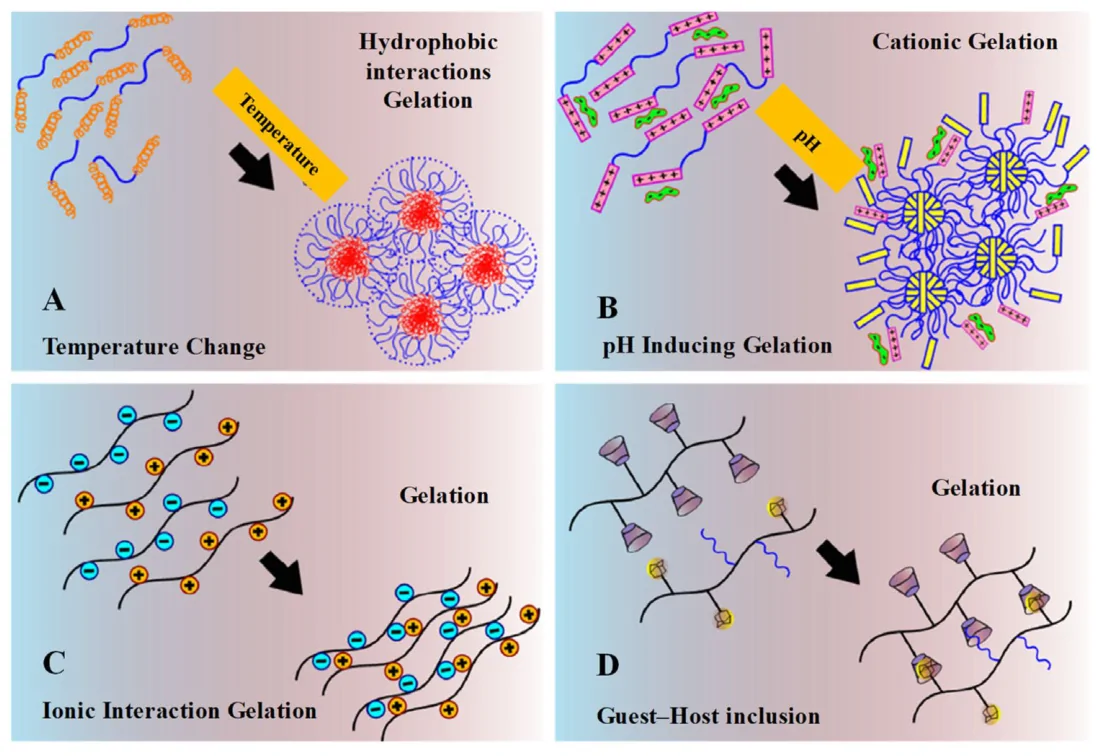
Major physical crosslinking mechanisms used in injectable hydrogels: (**A**) temperature-induced gelation caused by changes in hydrophobic interactions; (**B**) gelation driven by pH-induced protonation–deprotonation transitions in a cationic hydrogel; (**C**) gelation through ionic interactions; and (**D**) gelation through guest–host inclusion complexation between cyclodextrin groups and adamantane groups or PEG chains. The coloured shapes schematically represent the different polymer components and functional groups, while the black arrows indicate their transition from the un-crosslinked state to the gelled network. Reprinted from Ref. [38].

A key example of an injectable hydrogel that has physical crosslinking uses reversible and non-covalent interactions to create a gel without being chemically bonded. These hydrogels are based on thermoresponsive, pH-responsive, ionic, supramolecular, and self-assembling peptide systems as illustrated in Figure 5 [31]. Thermogelling hydrogels have been widely studied. These hydrogels are liquid at room temperature and rapidly change from sol to gel after injection at body temperature. This behaviour enables straightforward handling and administration while allowing spontaneous gel formation at the target site [34]. However, thermogelling systems often suffer from inadequate mechanical strength and their gelation properties can be affected by localised physiological conditions which can impact the stability, retention, and drug release of the gel [35]. Despite these limitations, thermoresponsive hydrogels still remain as one of the most extensively studied types of injectable systems since they do not need the use of external crosslinking agents. However, there are situations where the need for prolonged mechanical stability or sustained structural integrity is required, where chemically crosslinked hydrogels tend to perform better, although this comes with the expense of increased formulation complexity and stricter control over the gelation process [36,37].

Across the reviewed literature, subcutaneous injection and direct administration into the target tissue are the most frequently described routes for injectable hydrogels. Site-specific routes include intra-articular injection for musculoskeletal disorders and intratumoral or peritumoral injection for local cancer treatment. Intramuscular, intradermal, wound bed, and ocular administration are used more selectively according to the intended application. Selection of the administration route depends on the accessibility of the target tissue, the required hydrogel volume, local physiological and mechanical conditions, and the need to limit systemic drug exposure [32,36,37].

An alternative, or an addition, could be the application of pH-sensitive gels whose properties change upon interaction with different pH levels. These are types of materials which contain specific functional groups in their structure that allow them to interact with the drug and ensure retention of the drug in the gel [39,40]. Compared with thermoresponsive systems, pH-responsive hydrogels may provide greater control over drug retention through electrostatic interactions between the polymer network and charged therapeutic molecules. However, the pH-sensitive drug-delivery systems also have some limitations since their performance depends heavily on the local pH of body which may vary with different tissues and diseases. Because of the reversible nature of these physical interactions, physically crosslinked hydrogels are generally characterised by poor mechanical stability and reduced structural integrity relative to covalently crosslinked networks [41,42].

A second important class of injectable hydrogels is based on shear-thinning and self-healing behaviour. These hydrogels are crosslinked by reversible noncovalent or dynamic covalent interactions such as peptide self-assembly, hydrogen bonding, electrostatic attraction, host–guest interactions, and supramolecular complexation [43]. These dynamic interactions dissociate during injection under applied shear stress, and the hydrogel experiences temporary viscous flow. Upon removal of shear force, the interactions recover spontaneously, enabling the hydrogel network to recover at the designated site [44]. This property makes it possible to pre-shape these materials, load them with therapeutic agents, and inject them through fine-gauge needles without any permanent disruption of the network structure.

A third option is covalent crosslinking, which is more mechanically resilient. In these systems, injectable precursor solutions are delivered to the target site before local covalent crosslinking is initiated. Gelation can be achieved by photopolymerisation, enzyme-mediated reactions, Schiff-base chemistry, Michael-type addition, click chemistry, or other bioorthogonal reactions [45]. One of the most investigated methods is photopolymerisation, in which hydrogel precursors with photoinitiators are injected and then exposed to light to induce rapid crosslinking at the desired location. Covalent crosslinks, in contrast to reversible interactions, are generally considered irreversible, which allows the creation of mechanically stable and durable networks. However, when creating chemically crosslinked systems, there is a need for maintaining precise control over external variables or the use of specific initiators in order for the process of gelation to proceed successfully while maintaining biocompatibility and minimizing cytotoxicity [31].

Injectable hydrogels intended for clinical administration must be sterile. Sterilisation may be performed by steam treatment, gamma or electron beam irradiation, ethylene oxide exposure, or sterile filtration of low-viscosity precursor solutions followed by aseptic preparation. However, heat and radiation may cause polymer degradation or additional crosslinking, while chemical sterilant may leave toxic residues. These changes can affect gelation, swelling, mechanical strength, degradation, drug release, and the activity of incorporated therapeutic agents. Therefore, the sterilisation method must be selected according to the polymer composition and therapeutic payload, and the final sterilised formulation should be reassessed for sterility and functional performance [46].

Isotonicity is another important requirement for injectable hydrogels because marked differences between the osmotic pressure of the formulation and that of physiological fluids may cause pain, tissue irritation, cell damage, or changes in hydrogel swelling. Isotonicity can be adjusted using suitable concentrations of sodium chloride, phosphate-buffered saline, glucose, mannitol, or other biocompatible tonicity-adjusting agents. The final precursor formulation should have an osmolality close to that of physiological fluids, which is approximately 280 to 300 mOsm/kg, and this should be confirmed experimentally using an osmometer. Any tonicity adjustment must also be evaluated for its effects on gelation time, network structure, swelling, mechanical properties, and drug release [32].

### 3.3. Stimuli-Responsive Hydrogel Systems

**Figure 6:**
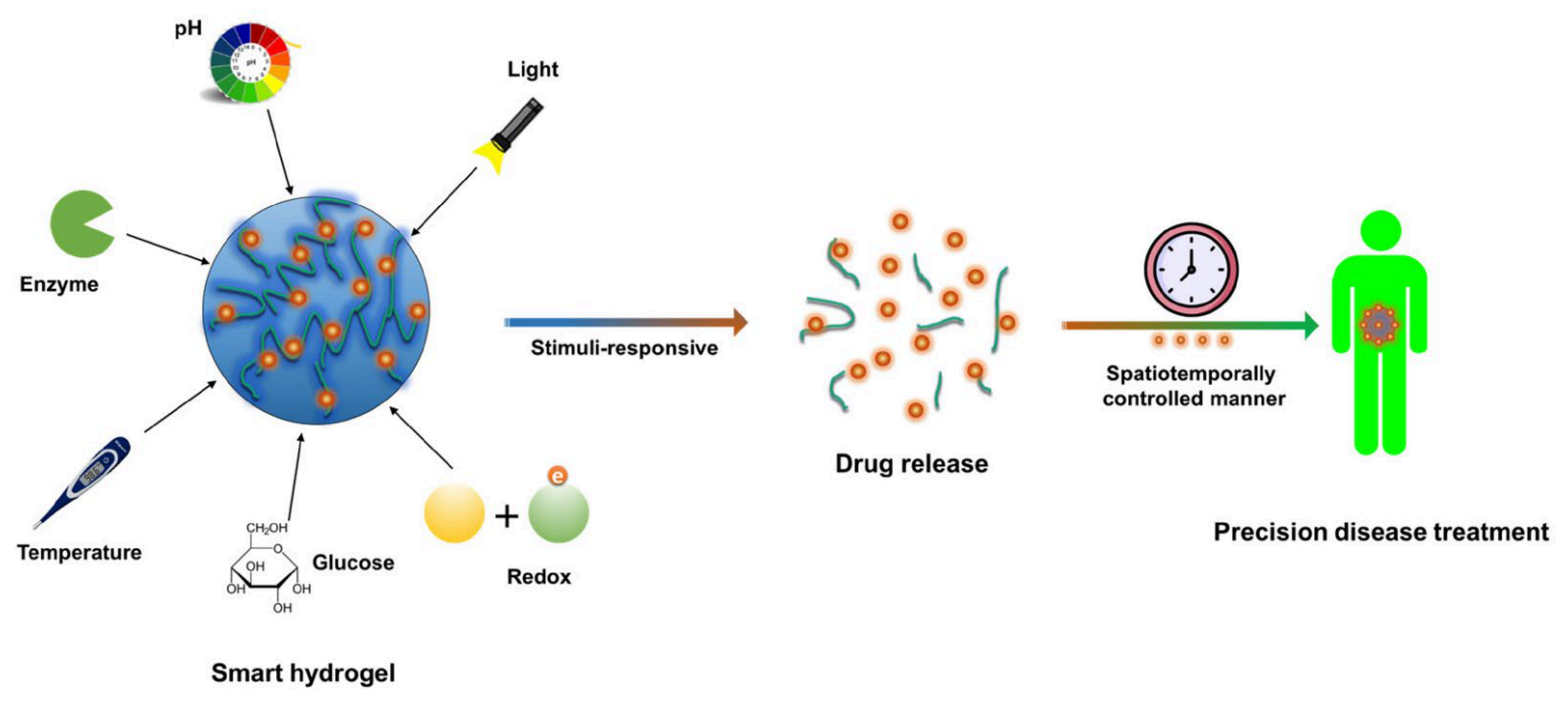
Summary of stimuli-responsive hydrogels and nanogels. The encapsulated drug can be activated for controlled release and targeted to the target site for precise disease treatment. Reprinted from Ref. [47].

Hydrogels can be formulated to respond to specific endogenous or applied stimuli. This allows the regulation of drug release by changes in the surrounding environment instead of passive diffusion or bulk degradation. Hydrogel swelling, degradation, and drug diffusivity can be altered in response to a defined stimulus through changes in polymer ionisation, molecular interactions, crosslinking density, or network integrity, providing a means to control the location and rate of therapeutic release [47,48]. Some of the most common triggers studied are pH, temperature, glucose, enzymes, redox, and light as shown in Figure 6.

A clear distinction should be made between stimuli that trigger hydrogel formation and stimuli that regulate drug release. For example, as discussed in Section 3.2, the temperature and pH may induce in situ gelation of an injectable precursor, but they may also act as environmental triggers that subsequently alter an established hydrogel network and modulate therapeutic release.

#### 3.3.1. Endogenous Stimuli-Responsive Hydrogels

The ionisable functional groups of pH-sensitive hydrogels, commonly weakly acidic groups such as carboxylic acids or weakly basic groups such as amines, can be protonated or deprotonated in response to changes in environmental pH. The electrostatic forces in the polymer matrix are altered by ionisation changes, leading to expansion or shrinking and thus changes in mesh size and drug permeability [49]. This mechanism is particularly relevant as pathological microenvironments such as tumours and inflamed tissues may have different pH levels compared to healthy physiological conditions, thus providing an inherent trigger for localised drug release [50]. However, the extent and spatial distribution of abnormal pH alterations can vary between patients, tissues, and disease stages, possibly resulting in inhomogeneous or inadequate drug release.

Glucose-responsive hydrogels are mainly based on phenylboronic acid (PBA)-based recognition mechanisms or mediated by glucose oxidase (GOx). Glucose competitively interacts with the boronic acid groups in the PBA-based systems, which may interfere with the reversible boronate–diol interactions, resulting in changes in the network crosslinking and swelling properties of the system, thus facilitating drug diffusion. Enzymatic oxidation of glucose in GOx-based systems generates gluconic acid and hydrogen peroxide, leading to localised chemical changes that can trigger pH-sensitive swelling or degradation of appropriately engineered polymer networks [51].

Redox-sensitive hydrogels exploit the differences in oxidative and reductive states between physiological and pathological microenvironments. Reduction-sensitive systems usually incorporate disulphide linkages that are cleaved in strongly reducing environments, particularly those with high levels of glutathione (GSH) resulting in network destabilisation and release of therapeutics [52]. Conversely, ROS-responsive hydrogels utilise oxidation-sensitive chemical groups that are cleaved or structurally modified upon exposure to elevated levels of reactive oxygen species leading to network degradation or changes in drug diffusion [53]. ROS-responsive systems are particularly relevant in cardiovascular injury because of the strong oxidative stress associated with myocardial ischaemia and ischemia-reperfusion injury. Therefore, ROS-sensitive injectable hydrogels have been investigated as localised therapeutic systems that can respond to the diseased microenvironment while retaining drugs at the damaged myocardial site [54].

#### 3.3.2. Exogenous Stimuli-Responsive Hydrogels

Exogenous stimuli can trigger externally controlled drug release, where temperature and light are some of the most studied initiators. Temperature-sensitive hydrogels are characterised by the changes in moisture content and the configuration of the network with the change in temperature. One of the most studied examples is poly(N-isopropylacrylamide) (PNIPAm) that remains hydrated and swollen below its lower critical solution temperature (LCST) but collapses into a shrunken network above the LCST as the polymer–water interactions are reduced and hydrophobic interactions dominate. This change alters the hydrogel mesh size and drug delivery, while the transition temperature can be tuned by changing the polymer formulation or by copolymerisation [34,55].

Light-responsive hydrogels possess photosensitive components which undergo structural alterations on being exposed to ultraviolet (UV), visible, or near-infrared (NIR) light. Structural alterations lead to the modification of the hydrogel matrix and consequently the controlled release of drugs due to photocleavage or photothermal response. In contrast to endogenous stimuli, light allows precise spatial and temporal control by facilitating the release of drugs only where and when irradiation occurs [39,56]. Although UV light is limited by poor tissue penetration and potential phototoxicity, NIR light penetrates deeper into tissues and has become the preferred option for localised drug delivery and cancer therapy [57].

The principal endogenous and exogenous stimuli used in responsive hydrogel systems, together with their effects on drug release and key practical considerations, are compared in Table 4.

## 4. Clinical Applications of Hydrogel-Based Drug Delivery

In many clinical contexts, having control of where and for how long a drug is exposed to tissue matters. This is because systemic administration is often the cause of off-target toxicity or a failure to maintain a therapeutic concentration at the targeted site [59,60]. Hydrogels have been explored as a platform that addresses this point and is suited to provide a localised and sustained exposure. These clinical applications of hydrogel-based drug delivery are discussed in four main areas: wound healing, cancer therapy, glucose-responsive insulin delivery, and musculoskeletal and inflammatory conditions. Each of these areas will be examined in terms of specific clinical rationale and evidence base [60,61,62].

### 4.1. Wound Healing and Diabetic Wounds

**Figure 7:**
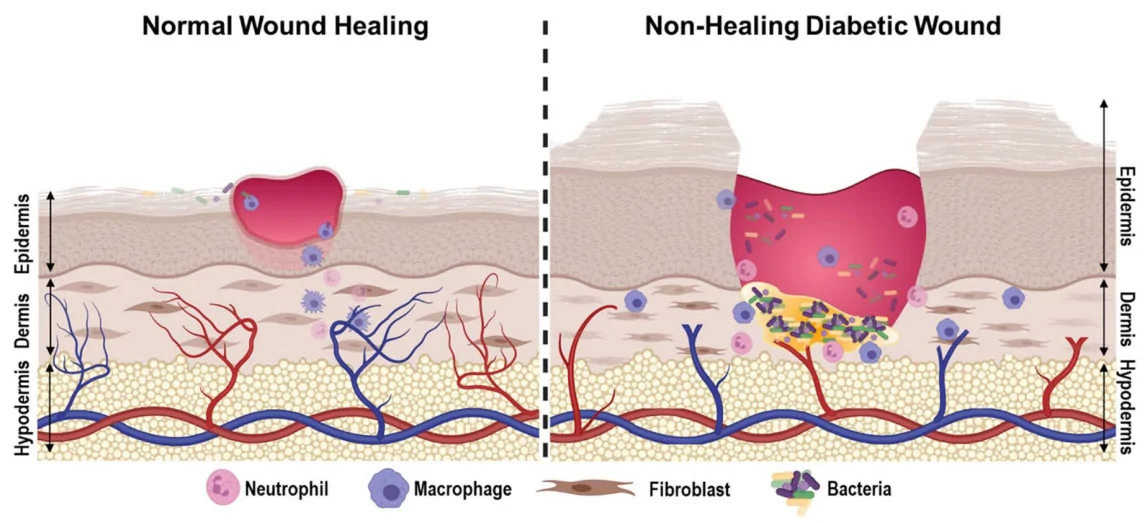
Pathophysiology of diabetic wounds. Diabetic wounds exhibit deregulated angiogenesis, chronically sustained sub-optimal inflammatory response, increased levels of reactive oxygen species, and persistent bacterial colonisation that often develops into a hard-to-treat biofilm. Reprinted from Ref. [64].

Chronic wounds have an impact on millions of people, with diabetic foot ulcers representing one of the most clinically significant and financially costly subtypes. Armstrong et al. reports that approximately 18.6 million people are affected by chronic wounds annually, and ulcers precede in up to 80% of lower extremity amputations in diabetic patients [63]. Impaired healing in diabetic wounds is driven by three converging factors: persistent infection, poor vascularisation, and unresolved inflammation (Figure 7) [64]. Systemic antibiotic therapy offers limited local tissue penetration in ischemic wounds and carries an additional risk, accelerated antimicrobial resistance [65].

Antimicrobial agents, including silver nanoparticles and levofloxacin, can be incorporated directly into hydrogel matrices, enabling controlled local release while potentially reducing systemic exposure [66,67]. Growth factors such as basic fibroblast growth factor (bFGF) can also be incorporated into hydrogel matrices to improve their local stability and provide sustained delivery, thereby supporting angiogenesis and tissue regeneration [68]. ROS-scavenging and immunomodulatory components can further reduce oxidative stress, regulate macrophage polarisation, and promote angiogenesis in diabetic wounds [69].

Hydrogel dressings, particularly alginate-based systems, can absorb wound exudate and maintain a moist environment that supports epithelial migration and wound closure. Their hydrated and conformable structures enable close contact with irregular wound geometries, while alginate matrices can also provide hemostatic support and act as local therapeutic carriers [70,71].

Recent reviews of self-healing hydrogel dressings highlight their ability to conform to irregular wound surfaces, maintain a moist environment, recover after mechanical disruption, and function as local therapeutic delivery systems. However, most advanced multifunctional formulations remain supported primarily by preclinical evidence [72].

**Figure 8:**
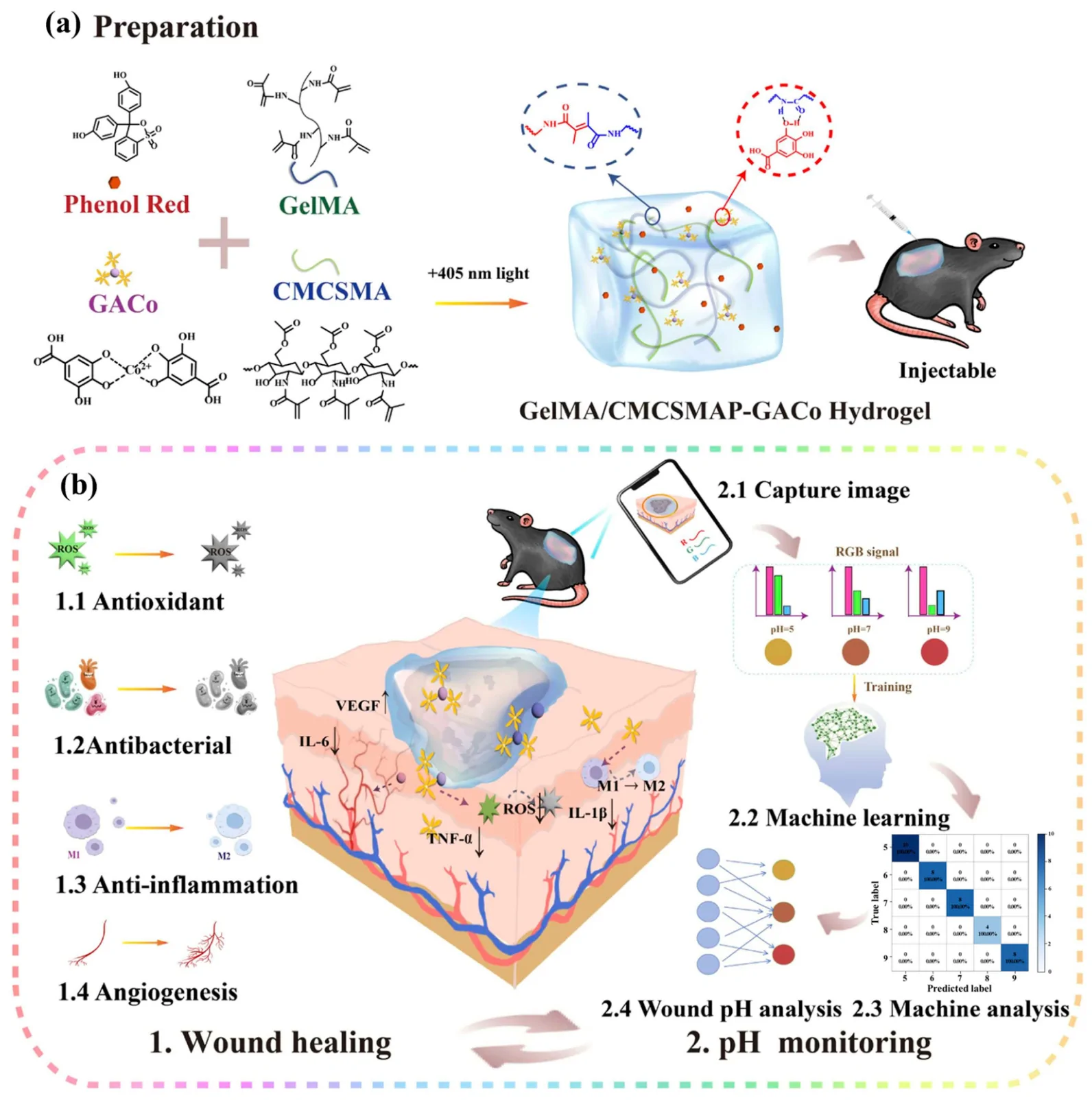
(**a**) Schematic illustration of the synthesis of GelMA/CMCSMAPGACo hydrogel; (**b**) schematic illustration of the potential application mechanism in diabetic wound healing. Reprinted from Ref. [74].

Hydrogel-based dressings, including silver-containing formulations, have progressed to clinical use with products that are commercially available. An example of this is Aquacel Ag, and this concept represents an established precedent for hydrogel wound management. However, the majority of evidence for advanced drug-loaded hydrogels in diabetic wounds remains at the preclinical stage, and large-scale randomised controlled trial data are still limited [73]. He et al. provides a systematic review of smart hydrogel dressings for diabetic wound management. The study highlights smart hydrogels and their role in responding to micro-environmental cues including ROS, pH, MMP-9, and glucose, enabling glucose release in response to these cues (Table 5). The paper also describes the emerging integration of machine learning with hydrogel-based sensors, exemplified by a GelMA-based system combining smartphone imaging with machine-learning algorithms that are able to assess wound pH with a 96% accuracy (Figure 8) [74].

Conventional gauze dressings do not have a mechanism that is able to respond to any of this biology. To be specific, they are unable to sense pH, degrade on cue, or release a growth factor at a controlled rate. That gap makes hydrogels a rational platform for chronic diabetic wound care [72,74].

### 4.2. Cancer Therapy

The central problem with systemic chemotherapy can be labelled as geography, where a drug meant for a tumour ends up circulating everywhere, and healthy tissue then pays the toxicity cost for treatment aimed elsewhere [75,76]. Many tumour sites are physically accessible for local treatment, particularly post-surgical resection cavities where any residual cancer cells can cause a relapse if left untreated [77,78].

Local hydrogel delivery can sustain therapeutically relevant drug concentrations at the tumour or postoperative site while reducing systemic exposure, making it a promising investigational strategy [79]. Chemotherapeutic agents such as doxorubicin, paclitaxel, and 5-fluorouracil can be incorporated directly into hydrogel matrices. Injectable and in situ-forming hydrogels can conform to irregular postoperative resection cavities and form local depots that release therapeutic agents over days to weeks. Biodegradable matrices may gradually break down during treatment, potentially avoiding the need for surgical removal. Together, these properties address several pharmacokinetic and practical limitations of conventional local cancer therapy [79,80,81,82].

Zhong et al. describes hydrogel local drug-delivery systems for postsurgical tumour management, covering both in situ and non-in situ platforms across a range of tumour types. The study demonstrates that injectable and in situ-forming hydrogels can deliver chemotherapeutic, phototherapeutic, and immunotherapeutic agents directly into resection cavities. This allows the achievement of high local drug concentrations while minimizing systemic drug exposure. Table 6 summarises the comparative advantages and disadvantages of each system type. The paper also acknowledges that despite preclinical promise, clinical translation is limited by challenges including hydrogel stability, absence of standardised safety protocols, and the gap between animal models and human physiology [78].

### 4.3. Diabetes: Glucose-Responsive Insulin Delivery

Type 1 diabetes and some insulin-requiring cases of type 2 diabetes require precise insulin dosing that responds to fluctuations in blood glucose throughout the day. Conventional subcutaneous insulin therapy may require multiple daily injections, contributing to treatment burden, while inappropriate dosing or timing can increase the risk of hypoglycemia [83,84]. These limitations have encouraged the development of closed-loop delivery systems that can respond autonomously to changing glucose concentrations. Glucose-responsive hydrogels are designed to increase insulin release as glucose levels rise and reduce release as glucose levels return towards the physiological range. Injectable hydrogel depots may also prolong insulin delivery and reduce injection frequency, although most systems remain at the preclinical stage [83,84,85,86,87].

In animal models, glucose-responsive hydrogel systems have demonstrated the ability to normalise blood glucose levels and maintain them within a physiological range over extended periods, providing proof of concept for the self-regulating mechanism. In a study conducted by Matsumoto et al., a synthetic protein-free boronic acid-based hydrogel was implanted subcutaneously in streptozotocin-induced type 1 diabetic mice. The results demonstrated glucose-responsive insulin release with a measurable reduction in glucose levels within 15 min of a glucose challenge. There was also sustained glycemic control over a period of three weeks and a reversal of elevated HbA1c levels without inducing hypoglycemia [86]. In other words, the hydrogel was able to adjust its own insulin output based on glucose levels without external dosing input. This is the core therapeutic promise of a closed-loop system.

Early translational work has begun to explore the feasibility of these systems in humans. However, this remains to be an emerging area with limited clinical trial data compared to the preclinical evidence base. To date, the closest clinical precedent is MK-2640, an insulin analogue and not a hydrogel platform. While there was an observed result, the trial was promptly discontinued due to insufficient potency in humans compared to preclinical models [88].

### 4.4. Musculoskeletal and Inflammatory Conditions

In the musculoskeletal field, conditions such as osteoarthritis, rheumatoid arthritis, and intervertebral disc degeneration represent major causes of chronic pain and disability worldwide [89,90]. Although NSAIDs, corticosteroids, and biological therapies can reduce pain and inflammation, prolonged systemic exposure may produce significant adverse effects. Intra-articular injection enables direct administration into the affected joint, but conventional soluble formulations may have limited residence within the synovial space and often provide only short-term therapeutic benefit [91,92]. Injectable hydrogels are therefore being investigated as local depots that can prolong joint residence, protect therapeutic payloads, and provide controlled release [91,93].

Intra-articularly injected hydrogel depots can sustain the local release of corticosteroids and prolong anti-inflammatory effect beyond what free drug injection achieves [94]. Growth factors like BMP have been incorporated into hydrogel systems in research settings to support cartilage repair, with the co-delivery of mesenchymal stem cells that has been recently explored in preclinical models. The viscoelastic properties of hydrogels are compatible with the mechanical environment of the joint, and biodegradable formulations eliminate the need for surgical removal [95]. Hyaluronic acid-based viscosupplementation provides an established clinical precedent for intra-articular polymer-based treatment, although its effectiveness and safety may depend on the formulation and administration schedule [96,97].

**Figure 9:**
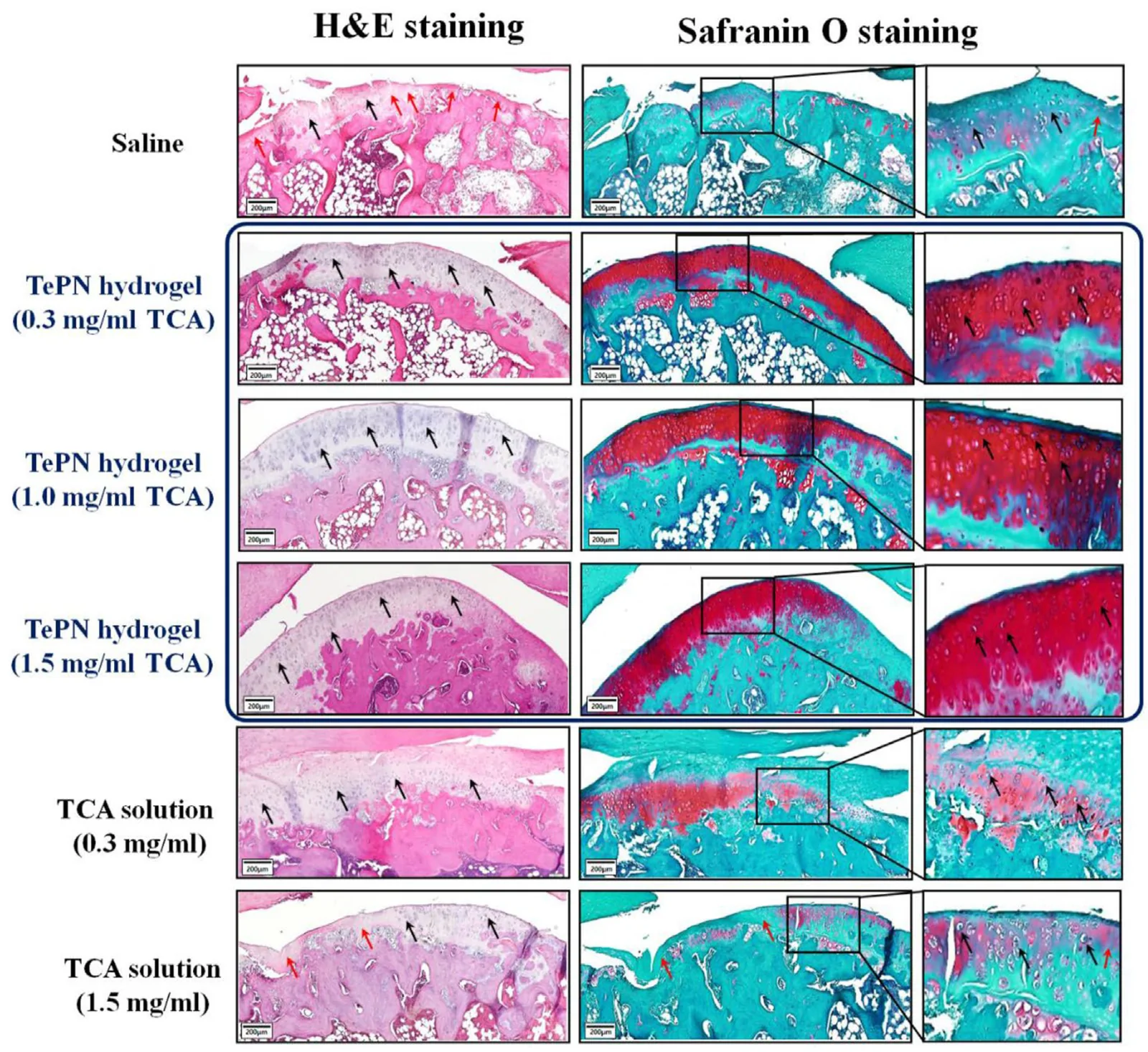
In vivo anti-inflammatory efficacy of TePN hydrogel systems after 8 weeks of treatment, evaluated using H&E and Safranin O staining. In the H&E-stained sections, black arrows indicate tissue containing chondrocytes, whereas red arrows indicate tissue without chondrocytes. In the Safranin O-stained sections, red staining indicates glycosaminoglycan-rich cartilage matrix. Scale bar = 200 μm. Reprinted from Ref. [98].

Preclinical studies suggest that intra-articular hydrogel delivery can prolong local drug residence and sustain anti-inflammatory effects compared with free-drug injection. Seo et al. evaluated a triamcinolone acetonide-loaded thermosensitive hydrogel in a rat osteoarthritis model. Following a single intra-articular injection, the hydrogel formed a depot and released the drug over approximately six weeks, whereas free triamcinolone acetonide was cleared from the joint within approximately one hour. Histological assessment at eight weeks showed better preservation of cartilage architecture, and intact chondrocytes and glycosaminoglycan staining in hydrogel-treated joints than in joints receiving the free drug, as shown in Figure 9. These findings support prolonged local delivery in a preclinical model but do not establish clinical efficacy or complete absence of systemic exposure [98].

## 5. From Experimental Formulation to Data-Driven Hydrogel Development

Building on the clinical applications discussed above, application-specific outcomes can serve as targets for data-driven optimisation. These include drug-release duration, wound closure, tumour response, glucose regulation, pain reduction, biocompatibility, and adverse effects. This section therefore defines the formulation variables that can be used as model inputs and the performance outcomes that can be used as prediction targets, linking hydrogel design directly with its intended therapeutic application.

The clinical performance of hydrogel-based drug-delivery systems does not rely solely on the selection of biomaterials but is also greatly influenced by the optimisation of formulation parameters that regulate their physicochemical properties and therapeutic efficacy [99]. Conventional hydrogel development relied on trial-and-error experimentation, involving repeated modifications of formulation parameters to identify the optimal conditions that achieve the desired drug-release kinetics and biological performance. However, this methodology is associated with limitations including high resource and time consumption, which has triggered the development of computational approaches such as machine-learning models. These models facilitate accurate predictions of hydrogel characteristics and the optimisation of formulation variables [100,101].

### 5.1. Key Formulation Variables

Formulation variables including hydrogel materials, polymer type and concentration, crosslinking strategy and density, and the physicochemical characteristics of the encapsulated drug collectively influence the hydrogel’s mechanical strength, drug-loading capacity, and drug-release kinetics [102]. The polymeric nature of hydrogels allows for precise tunability; for example, polymer composition, concentration, and crosslinking can all be altered to influence network degradation and swelling as well as drug-release kinetics [102,103,104].

Consequently, researchers have attempted to utilise synthetic polymers as alternatives in hydrogel fabrication including polyethylene glycol (PEG), polyacrylamide (PAAm), poly(lactic-co-glycolic acid) (PLGA), and poly(N-isopropylacrylamide) (PNIPAm). Synthetic polymers have gained increasing attention in hydrogel-based drug delivery due to their highly tunable properties, in turn allowing precise control over hydrogel network, mechanical strength, degradation behaviour, and drug-release profiles [105]. Unlike many natural polymers, synthetic polymers provide greater chemical versatility, allowing incorporation of stimuli-responsive behaviours. For example, PNIPAm can undergo temperature-induced phase transitions, as well as pH-responsive synthetic polymer systems that can be engineered to release the encapsulated drug in response to surrounding pH changes [106]. Table 7 compares the applications and advantages of natural and synthetic polymers in hydrogel systems [107].

**Figure 10:**
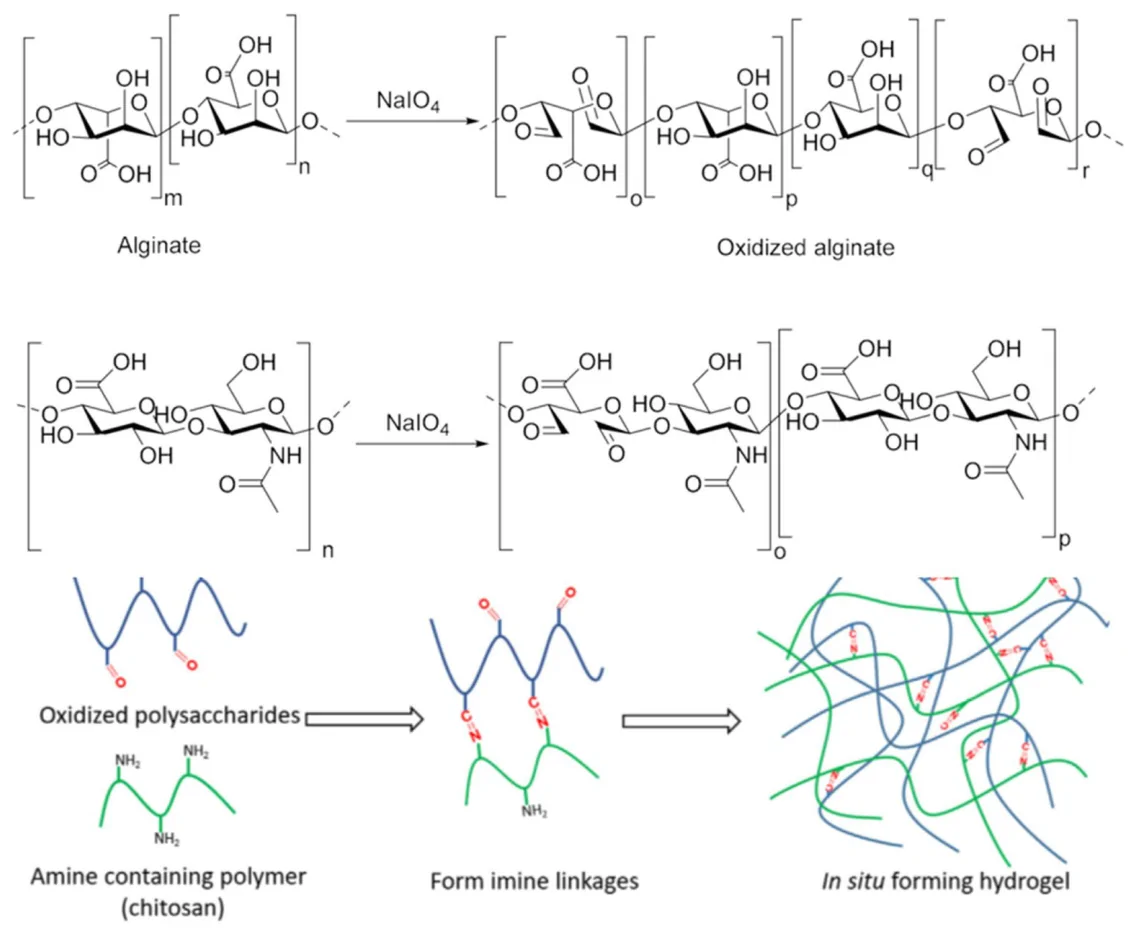
Schiff-base reaction. Periodate oxidation of polysaccharides exemplified on two of the most representative examples, i.e., alginate and hyaluronic acid, and a typical representation of an in situ-forming hydrogel via Schiff-base reaction. Reprinted from Ref. [112].

In addition to polymer selection, polymer concentration is another critical formulation variable that influences the therapeutic performance of hydrogel drug-delivery systems. Increasing polymer concentration promotes increased polymer network density, contributing to a more compact 3D matrix with reduced pore size and decreased swelling capacity. Consequently, this results in reduced molecular diffusion within the hydrogel matrix contributing to reduced drug mobility and controlled drug-release profiles [108]. Moreover, crosslinking strategy and crosslink density also influence the functional properties of hydrogel systems and their drug-release kinetics [109]. Chemically crosslinked hydrogels, including PEG-based networks formed by Michael addition or photopolymerisation, contain permanent covalent bonds that maintain highly stable polymer networks joined by covalent bonds [109]. Increased crosslinking density restricts polymer chain mobility and reduces hydrogel mesh size, contributing to increased diffusional resistance and improved drug retention. This is particularly useful for drug-delivery systems that require sustained controlled release of therapeutic agents over a prolonged time [110]. In contrast, dynamic crosslinking strategies utilise biologically cleavable bonds that respond to biological stimuli to facilitate regulated drug release [111]. For example, Schiff-base crosslinked chitosan hydrogels use imine bonds between aldehyde groups and chitosan amines to form reversible covalent linkages that can undergo hydrolysis in acidic environments resulting in the controlled pH-responsive diffusion of therapeutic agents (Figure 10) [112]. Importantly, ML models for drug-release prediction should incorporate not only hydrogel network characteristics but also descriptors representing drug–polymer interactions. Relevant drug-related input features may include molecular weight, molecular size, charge, pKa, aqueous solubility, hydrophobicity (e.g., logP), and hydrogen-bonding capacity. These descriptors can be combined with polymer characteristics such as polymer charge, functional groups, hydrophilicity, and crosslinking density to represent the compatibility between the drug and the hydrogel network. For example, electrostatic attraction between oppositely charged drugs and polymers may increase drug retention and slow release, whereas electrostatic repulsion may facilitate release. Similarly, hydrophobic interactions and hydrogen bonding can influence drug partitioning and mobility within the polymer network. Where experimental data are available, interaction-related measurements such as drug–polymer binding affinity, partition coefficient, or encapsulation efficiency can also be included as model features. Incorporating these interaction descriptors alongside structural parameters such as mesh size, swelling and degradation would allow ML models to better distinguish whether observed release behaviour arises primarily from network-controlled diffusion or from specific drug–polymer interactions. Evidently, successful hydrogel formulation requires simultaneous optimisation of a variety of formulation variables to achieve the most effective and predictable drug-delivery system.

### 5.2. Key Performance Outcomes: Swelling, Degradation, Mechanical Properties, and Drug-Release Profile

**Figure 11:**
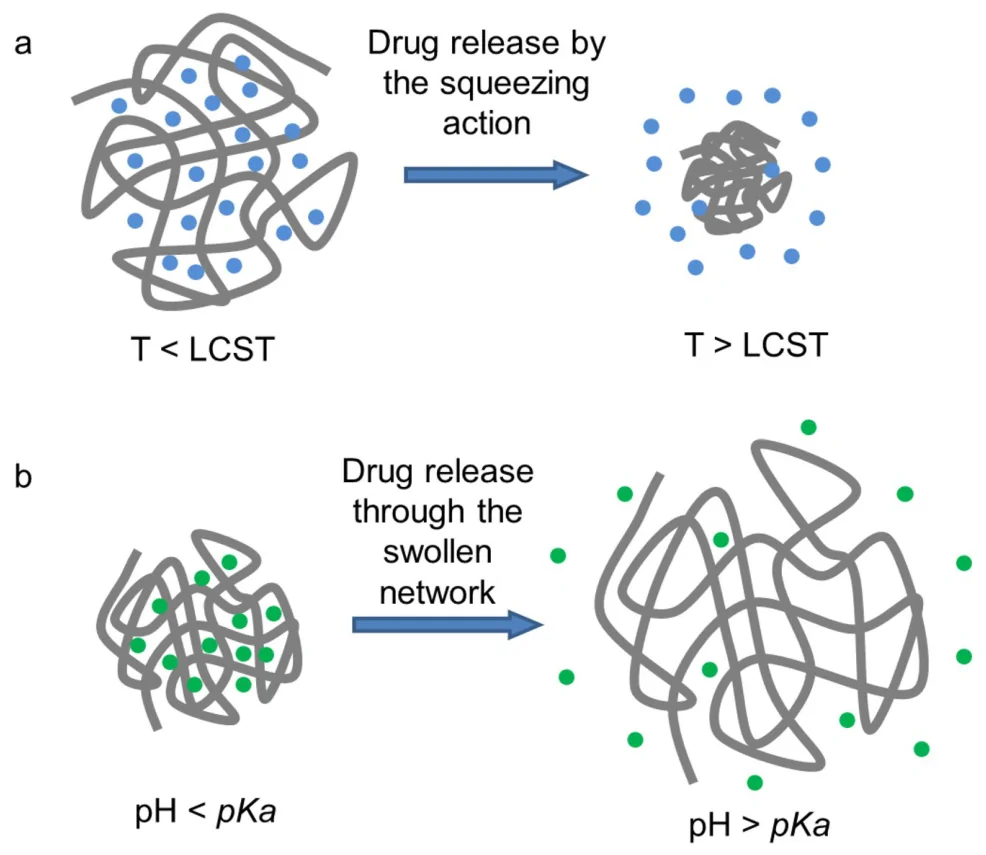
Schematic illustration of drug release from temperature-sensitive and pH-sensitive hydrogels. (**a**) Drug release from a temperature-sensitive hydrogel. Below the LCST (T < LCST), the polymer network is swollen and retains drug molecules, whereas above the LCST (T > LCST), the hydrogel collapses and releases the drug through a squeezing action; (**b**) drug release from a pH-sensitive hydrogel. At pH < pKa, the polymer network remains collapsed and retains drug molecules, whereas at pH > pKa, ionisation of functional groups causes swelling of the hydrogel network, leading to drug release. The blue and green dots represent drug molecules in panels (**a**) and (**b**), respectively. Reprinted from Ref. [113].

**Figure 12:**
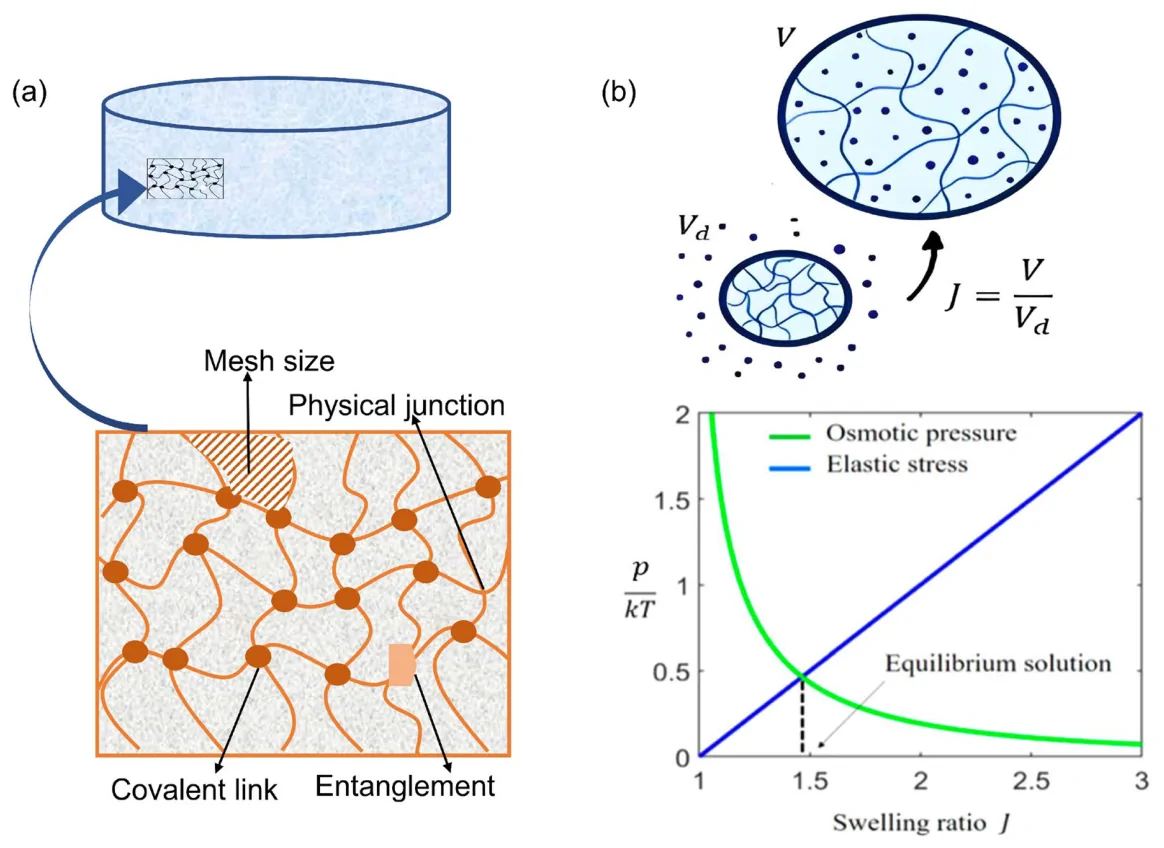
Structural chemistry of a hydrogel: (**a**) structure of a hydrogel at the molecular level; (**b**) correlation of the swelling ratio of the hydrogel with osmotic pressure and elastic stress. Reprinted from Ref. [114].

Swelling and mesh size are central determinants of drug transport. As illustrated in Figure 11 and Figure 12, water uptake can expand the hydrogel network and facilitate molecular diffusion, whereas stimulus-induced network collapse may promote drug release through a squeezing mechanism. These behaviours reflect the balance between osmotic pressure and network elasticity [113,114].

Several key performance outcomes are evaluated following hydrogel fabrication to determine the suitability of the resulting system. Swelling is a central parameter arising from hydrophilic interactions between the polymer chains and surrounding water molecules, while covalent crosslinks, physical junctions, and chain entanglements generate elastic resistance that prevents uncontrolled expansion. The equilibrium swelling state therefore reflects the balance between the osmotic pressure promoting water uptake and the elastic stress opposing network expansion, as shown in Figure 11. Hydrogel mesh size is a key determinant of this behaviour [113]. Increased water uptake promotes polymer-chain relaxation and enlarges the mesh size, creating wider diffusion pathways that facilitate drug transport and may accelerate drug release [113,114]. As shown in Figure 11, drug release may occur through the expansion of a swollen network or through the squeezing action associated with network collapse in response to changes in pH or temperature. Together, Figure 11 and Figure 12 demonstrate how network structure, swelling, and stimulus-responsive volume changes govern drug transport and release from hydrogels.

Degradation behaviour is a direct outcome that influences drug-release kinetics and biological performance. The degradation of biodegradable hydrogels is governed by the crosslinking strategy of the matrix [115]. Natural polymers, including gelatin and collagen, are enzymatically degraded by matrix metalloproteinases and collagenases that allows biomimetic remodelling of the hydrogel matrix, making them especially useful in tissue regeneration applications, such as wound healing [116], whereas synthetic PEG-based hydrogels have a more stable covalent network structure resulting in slower matrix degradation and consequently extended drug release [117]. Such extended-release systems would be suitable for ocular drug delivery applications, such as anti-VEGF agents, specifically ranibizumab, in turn reducing the frequency of intravitreal injections required for neovascular age-related macular degeneration [118].

Mechanical properties represent performance parameters in hydrogel-based drug-delivery systems as they determine the ability of the polymer to withstand physiological stress and maintain structural integrity [119]. Properties such as compressive modulus, tensile strength, Young’s modulus, and viscoelasticity can be modified by adjusting the formulation parameters mentioned earlier [120]. Increasing polymer content and crosslinking density enhances hydrogel mechanical properties, including compressive modulus and tensile strength by increasing polymer density and reducing polymer chain mobility [121]. For example, the mechanical properties of GelMA hydrogels can be tuned by adjusting the polymer concentration, degree of methacryloyl substitution, and crosslinking conditions. Increasing GelMA concentration or the availability of methacryloyl groups generally increases crosslink density and elastic modulus. However, GelMA concentration should be expressed as a percentage, such as % *w*/*v*, whereas mechanical modulus should be reported in Pa or kPa; these measurements should not be treated as equivalent. For load-bearing applications, the formulation should be optimised to balance mechanical support with swelling, degradation, drug transport, and biological compatibility [122,123].

### 5.3. Limitations of Repeated Experimental Optimisation Through Trial-And-Error Methods

Conventional hydrogel-based drug-delivery systems relied on trial-and-error methods by adjusting individual formulation variables until the desired physicochemical properties and drug-release profile were achieved [124]. Albeit, as the number of formulation variables incorporated in the hydrogel system increases, this method becomes increasingly challenging, as the interactions between these variables are usually nonlinear and difficult to predict using conventional methods [124]. For instance, increasing the polymer concentration in GelMA hydrogels from 5% to 15% increases the density of the network along with the compressive modulus and tensile strength but it reduces the swelling ratio and subsequently the drug diffusion rate [125]. Similarly, increasing the degree of methacrylation in GelMA hydrogels increases mechanical stability but reduces the drug-release kinetics [125]. Accordingly, trial-and-error strategies are inefficient for optimising hydrogel-based drug-delivery systems, as they do not adequately capture complex relationships between formulation variables and system performance. Another limitation of this methodology is the exponential growth in experimental combinations required as additional formulation variables are introduced. Consequently, exhaustive testing of all possible formulation combinations becomes resource and time intensive and becomes increasingly impractical to implement [124]. Moreover, optimising one parameter independently has shown to produce suboptimal formulations as improvements in one performance outcome occurs at the expense of another outcome. This complex interplay between formulation variables and performance outcomes coupled with the limitations of conventional hydrogel methodologies underscore the importance of computational approaches capable of simultaneously modelling multiple interacting variables [126].

Together, these formulation variables and performance outcomes establish the model input–target relationships used in the machine-learning framework discussed in Section 6.

### 5.4. Preparation of Hydrogel Experimental Data for ML-Based Prediction and Optimisation

To successfully implement machine learning for hydrogel-based drug-delivery systems, large high-quality datasets that accurately capture the relationships between formulation variables and performance outcomes are required. Each hydrogel formulation is represented by a set of input features that describe its material composition and fabrication parameters, along with the experimentally measured output variables including swelling ratio, degradation rate, compressive modulus, encapsulation efficiency, and drug-release kinetics [127]. The raw data must undergo systematic preprocessing to improve data quality, which includes data cleaning and normalisation. Subsequently, feature selection will be performed to identify the formulation variables that most significantly influence performance outcomes. For instance, in GelMA hydrogels, this would include GelMA concentration, degree of methacrylation, photoinitiator concentration, drug-loading percentage, and crosslinking density. When necessary, feature engineering will be performed to introduce new variables that better represent the mechanisms influencing performance outcome to improve the predictive accuracy. For example, rather than utilising individual formulation parameters, researchers can engineer features such as GelMA concentration-to-photoinitiator ratio, crosslinking energy, or polymer-to-drug ratio [128]. As two GelMA formulations both containing 10% GelMA can exhibit different swelling ratios and drug-release kinetics depending on the degree of methacrylation and other factors [129], combining experimental variables into biologically meaningful features will allow machine-learning models to better capture the complex relationships between formulation parameters and hydrogel performance.

To establish a more systematic link between hydrogel physicochemical characteristics and ML feature engineering, Table 8 maps key experimentally measurable hydrogel properties to their corresponding quantitative model representations. These features may be incorporated directly from experimental measurements or constructed as derived features that preserve physically meaningful relationships between formulation variables.

As shown in Table 8, the representation of hydrogel characteristics as ML features should reflect their underlying physicochemical roles. Crosslinking parameters can be used to describe network architecture because increasing crosslink density generally reduces mesh size and restricts molecular diffusion [108,109,110]. Swelling measurements provide information on water uptake and network expansion and can therefore be represented by equilibrium swelling ratio or time-dependent swelling values [113,114]. Similarly, degradation can be represented by mass loss, degradation rate, or measurements at defined time points, providing quantitative information on matrix persistence and degradation-controlled release [115,116,117]. Mechanical characteristics, including compressive modulus, tensile strength and viscoelastic parameters, provide quantitative descriptors of structural performance [119,120,121,122,123]. Importantly, feature engineering can also capture relationships between variables rather than considering each property independently. For example, the ratio or relationship between drug molecular size and hydrogel mesh size is physically relevant because diffusion becomes increasingly restricted as drug size approaches the dimensions of the hydrogel mesh [8,16,22,23]. Thus, the selection and engineering of ML features should be guided by the physical mechanisms governing swelling, degradation, mechanical behaviour, and drug transport.

## 6. Machine Learning Across the Hydrogel Development Pipeline

The development of hydrogels for drug delivery is not a singular experimental effort. It progresses through five unique stages: material selection, formulation and synthesis, biophysical characterisation, drug-release modelling, and clinical response prediction [130].

The advancement of medical hydrogel technology is transitioning from conventional trial-and-error methodologies toward more efficient, data-informed frameworks. These intricate three-dimensional polymeric networks demonstrate properties influenced by non-linear interactions among their composition, synthesis processes, and the physiological milieu. In the past, traversing this high-dimensional domain required substantial resources and relied heavily on chemical intuition. With advances in computational resources, however, this can now be treated as a high-dimensional information processing problem. This is the reason machine learning has, in recent years, been used across different stages of hydrogel production, though not evenly [131].

Drawing on established applications of machine learning and deep learning in complex data-analysis settings [132,133,134], this review examines the integration of four fundamental machine-learning categories: (i) regression, (ii) feature selection, (iii) optimisation, and (iv) classification, across the five stages of the hydrogel lifecycle. We intentionally selected these four categories as they represent the fundamental principles of data-driven material design. Feature selection determines the key characteristics. Regression and classification offer predictive frameworks for continuous and discrete outcomes, respectively. Optimisation facilitates methodical exploration of the high-dimensional design space. This framework is not random; it embodies a mathematical classification that has demonstrated efficacy across other data-intensive fields, and we contend that it is overdue for systematic implementation in biomedicine. This expertise in several fields enables us to assert that hydrogel creation should not depend on trial and error but rather be approached as a predictive modelling challenge. This section’s contribution is a systematic map of four machine-learning problem types (feature selection, regression, classification, and optimisation) against each stage of hydrogel development, showing where each is applied and where it is not. Existing studies treat the four categories in isolation: one paper trains a regression model, another performs an optimisation, a third selects relevant features and stops there. The map makes a further pattern visible: even where a tool is applied at one stage, its output rarely feeds the next. Feature selection at the material selection stage should narrow the variables passed into optimisation at the synthesis stage; regression at the drug-release stage should feed a classifier that evaluates predicted release against clinical thresholds. In the literature reviewed, neither connection is established. Since the individual tools already exist at each stage, what remains is to chain them, and that chaining, rather than any single new model, is the argument this map is built to support. Table 9 lays out this mapping in full before we walk through it stage by stage, naming the current applications, the gaps, and the most promising future direction at each stage.

### 6.1. Stage 1: Material Selection

Choosing the right polymer, crosslinker, and additive combination is the first decision in hydrogel development and one of the highest stakes. A poor choice here propagates through every downstream step. ML enters as a screening tool, filtering a large combinatorial space before any experiment is run.

At the material-selection stage, Liao et al. combined protein-sequence mining with nine regression models to guide the design of bioinspired adhesive hydrogels [135]. The models were developed using an experimental dataset of 180 hydrogels and evaluated using a 90/10 training–test split, 10-fold cross-validation, and subsequent experimental testing. This approach produced a hydrogel with underwater adhesion exceeding 1 MPa. However, the predicted endpoint was adhesive strength rather than drug-release performance, and the model was not validated using clinical data.

### 6.2. Stage 2: Synthesis and Formulation

Once materials are chosen, synthesis introduces a second layer of variables: component concentrations, reaction temperature, initiator proportion, crosslinker-to-monomer ratio, and curing time. These interact nonlinearly. Small changes in initiator concentration can shift crosslink density enough to alter release kinetics by an order of magnitude.

Xu et al. applied all four ML problem types within a single study on polyacrylamide/alginate hydrogels. A classification model separated gelation regimes, feature importance scores identified AM concentration, ammonium persulfate, and N,N-methylene bisacrylamide as dominant synthesis variables, a regression model predicted mechanical properties, and Bayesian optimisation searched the seven-dimensional synthesis space to identify a superior formulation [137]. This is the closest existing study to the unified pipeline proposed here, although it was conducted for flexible electronics rather than drug delivery, meaning the clinical translation of its methodology remains untested.

Seifermann et al. trained Gaussian process models on data from over 900 photodegradable hydrogel formulations and used Bayesian optimisation with an expected improvement acquisition function to iteratively improve degradation response properties [138]. Most hydrogel ML datasets contain tens to low hundreds of observations; this study’s scale is notable and demonstrates that active learning loops can extract substantial gains even when individual experiments are slow.

### 6.3. Stage 3: Physicochemical Characterisation

Characterisation encompasses measurement of mechanical stiffness, swelling ratio, gelation temperature, porosity, and rheological behaviour. These continuous outputs are naturally regression problems. Neural networks and gradient-boosting models have been trained to predict storage modulus (G′) and loss modulus (G″) from formulation descriptors, with deep learning achieving competitive accuracy when dataset sizes are sufficient [141].

Feature selection serves a diagnostic function here. Xu et al. found that crosslink cycle number and freeze–thaw conditions dominated tensile properties in PVA hydrogels [142]. Knowing which process parameters control stiffness is more useful than knowing a model’s prediction accuracy; it redirects future experimental effort.

Classification and optimisation at Stage 3 connected directly to clinical acceptability criteria remain underexplored. No published study has classified characterisation outcomes against tissue-specific mechanical requirements, for example, whether a measured stiffness falls within the range required for cartilage versus wound-healing applications. This is a gap.

### 6.4. Stage 4: Drug-Release Modelling

Drug-release prediction represents the most extensively researched application of machine learning within this particular field. The inquiry concerning the proportion of drug released at a specified time under certain conditions constitutes a regression problem characterised by both continuous inputs and outputs.

AL-Rajabi et al. conducted a comparative analysis of six regression models pertaining to antibiotic release from temperature-responsive hydrogels, demonstrating that ensemble methods consistently surpassed individual models in performance [144]. Gaussian process regression is especially advantageous in this context as it provides calibrated uncertainty estimates alongside its predictions, a feature of practical significance when the release data are limited at intermediate time intervals. An uncertain prediction should not be regarded as a failure; rather, it constitutes valuable information that can inform subsequent experimental endeavours.

The process of feature selection at Stage 4 effectively translates model outputs into chemically actionable recommendations. Zhang et al. showed this sequence outside the drug delivery context: XGBoost importance scores identified reaction temperature, crosslinker concentration, and initiator proportion as the predominant variables in a hydrogel sorbent system, then fed these findings into a Bayesian optimisation loop [139,146]. The application was heavy-metal removal, not drug release, but the pattern is the same one this pipeline argues for. A closer example exists. Song et al. built a two-stage model for PLGA microsphere drug release: a classifier first predicts whether a formulation falls into slow-release behaviour, then that prediction becomes an input feature for a regression model estimating the full release curve [147]. XGBoost gave the lowest error among the models tested, and SHAP values pointed to drug and polymer molecular weight as the dominant features. This is the chaining Section 1 keeps asking for: classification feeding regression, not run side by side.

Classification at Stage 4 pertains to binary clinical inquiries: does the formulation fulfill a specified 24 h release criterion? Will the burst release surpass a therapeutic threshold? These inquiries cannot be directly addressed by regression models without implementing post hoc thresholding, yet they represent the fundamental questions posed by clinicians in practice.

### 6.5. Stage 5: Clinical Outcome Prediction

At Stage 5, we find a notable silence in the literature. There is a clear gap when it comes to using machine learning (ML) to predict how effective hydrogel formulations will be in real-life therapeutic settings, including how tissues respond or what patient outcomes might look like. According to Li et al., predicting clinical outcomes is an area ripe for exploration, and while there are AI applications in related biomedical fields, we still lack solid evidence specifically for hydrogel drug delivery. This highlights a pressing need for more research to delve into how AI can be integrated to better predict clinical outcomes in the context of hydrogel drug-delivery systems.

This inequality is really rooted in the system itself. Gathering clinical outcome data can be quite costly and comes with a host of ethical challenges. Plus, different institutions often work in isolation, and the timing for measurements does not really sync up with how machine learning evolves. We know there is a significant gap between predictions made in laboratory tests (in vitro) and how things actually perform in the body (in vivo). Factors such as how proteins stick to surfaces, how enzymes break things down, immune responses, and even physical stress can all change how hydrogels behave. Unfortunately, these effects cannot be identified simply by testing in a saline solution. Models that rely only on laboratory data often struggle to accurately predict real-world outcomes unless they also incorporate actual in vivo data or are guided by established scientific principles.

Han et al. come closest, using interpretable ML to optimise hydrogel scaffold properties, including modulus, swelling, and porosity, toward anti-inflammatory macrophage polarisation [140]. It is a real step toward Stage 5, but it stops the cell-level immune response rather than patient outcomes.

All four ML problem types are identified as gaps at Stage 5. Regression models predicting healing outcomes, classification models screening for biocompatibility, feature selection identifying which formulation variables drive clinical response, and optimisation targeting patient-level therapeutic endpoints do not exist in a published, validated form for hydrogel drug delivery. This is the most significant frontier the field faces and the one where collaboration between ML researchers, materials scientists, and clinical investigators is most urgently needed.

### 6.6. Critical Comparison of Existing Machine-Learning Studies

The available studies differ substantially in their datasets, hydrogel systems, and modelling objectives. Dataset sizes range from sparse experimental swelling measurements to 169 bioink measurements, 180 adhesive-hydrogel formulations, 350 literature-derived PVA records, and more than 900 photodegradable hydrogel formulations [135,136,137,138,139,140,141,142,143]. Conventional models, including artificial neural networks and XGBoost, have been used to predict rheological and mechanical properties [138,141,142]. Recent studies have extended ML towards biological applications, including wound monitoring and healing [139], macrophage polarisation [140], antitumour efficacy [143], and the discovery of nucleoside hydrogels with antibacterial, anti-inflammatory, and tissue-regenerative activity [145]. Direct comparison of model accuracy remains difficult because the studies used different materials, endpoints, dataset sizes, and evaluation metrics.

The validation strategies also varied considerably. Several studies relied mainly on internal train-test splitting or cross-validation, which evaluates performance within the available dataset but provides limited evidence of transferability to other hydrogel systems [141,142,143]. Stronger approaches combined computational predictions with experimental testing, including experimental validation of adhesive hydrogels [135], closed-loop optimisation of photodegradable formulations [137] and prospective active-learning optimisation of drug-release profiles [144]. Li et al. extended validation to in vitro testing and mouse periodontitis models, but this remains preclinical evidence rather than proof of clinical efficacy [145]. Overall, most models were developed using a single hydrogel family; few used independent external datasets, and none demonstrated patient-level clinical validation.

## 7. Translational Challenges and Future Directions

A major obstacle to the translation of machine learning in hydrogel research is the limited availability of large, high-quality and standardised datasets. Existing studies commonly rely on relatively small datasets generated using different hydrogel materials, synthesis conditions, drug-loading procedures, and characterisation methods. Differences in measurement units, experimental protocols, and reporting practices make it difficult to combine data across studies and may produce models that perform well only on the datasets used for their development [148,149,150]. Establishing shared reporting standards and accessible multi-institutional databases, including unsuccessful formulations and negative experimental findings, would improve dataset diversity, reduce publication bias, and support the development of more generalisable models. The principal translational barriers and proposed strategies are summarised in Table 10.

The validation of ML predictions presents another important challenge, particularly with respect to data splitting and leakage control. Data leakage occurs when information from the test set, either directly or indirectly, influences model training, resulting in overly optimistic estimates of predictive performance. This risk is particularly relevant for hydrogel datasets because multiple observations may originate from the same experimental batch, study, material family, or closely related formulations. If such correlated observations are randomly divided between training and testing sets, the model may effectively be evaluated on samples that are highly similar to those it has already encountered during training, rather than on genuinely independent formulations. Therefore, data splitting should be performed at an appropriate group level, such as by experimental batch, study, material family, or formulation, so that related observations remain within the same partition. Data preprocessing steps, including normalisation, feature selection, and imputation, should also be fitted using the training data only and subsequently applied to validation and test data to avoid indirect leakage. Where sufficient data are available, independent external datasets and prospective experimental testing should be used to provide a more reliable assessment of model generalisability [158,159]. Translating predictions from in vitro experiments into clinical outcomes is particularly difficult because hydrogel behaviour in vivo is influenced by protein adsorption, enzymatic degradation, immune responses, mechanical stress, and patient-specific physiological conditions. Models developed exclusively from simplified laboratory experiments may therefore fail to predict biological or therapeutic performance unless they incorporate in vivo evidence or established mechanistic principles [160]. The minimum reporting and validation requirements for future ML-guided hydrogel studies are presented in Table 11.

Safety, biocompatibility, degradation, and regulatory requirements must also be incorporated into ML-guided hydrogel optimisation. A formulation with favourable mechanical properties or drug-release kinetics may remain unsuitable for clinical use if it produces toxic degradation products, causes an undesirable immune response or cannot be manufactured consistently. Future optimisation frameworks should therefore evaluate multiple outcomes simultaneously, including drug release, mechanical stability, degradation, cytotoxicity, and biological response [161,162]. Greater integration of hydrogel composition, experimental characterisation, and biological outcome data could eventually support closed-loop development systems in which ML recommends formulations, experimental results update the model, and clinically relevant candidates are progressively prioritised.

## 8. Conclusions

Hydrogels offer a versatile platform for controlled and localised drug delivery because their composition, swelling, degradation, and mechanical properties can be adjusted to meet different therapeutic requirements. Natural and synthetic hydrogels have demonstrated potential across wound healing, cancer therapy, diabetes, and musculoskeletal or inflammatory conditions, particularly when injectable or stimuli-responsive systems are used to provide sustained and site-specific treatment. However, hydrogel performance depends on complex interactions among the selected materials, crosslinking conditions, drug characteristics and physiological environment. This complexity limits the effectiveness of conventional trial-and-error development and contributes to difficulties in achieving reproducible formulations with predictable drug-release behaviour.

Machine learning offers a practical route for improving the efficiency and precision of hydrogel development by predicting material properties, identifying influential formulation variables and prioritising the most promising experiments. Regression, classification, Bayesian optimisation, active learning, and explainable AI can collectively support the transition from isolated experimental studies towards an integrated and data-driven development process. Nevertheless, ML should complement rather than replace experimental and clinical validation. Successful translation will require larger and standardised datasets, independent model validation, assessment of biocompatibility and degradation, and early consideration of manufacturing and regulatory requirements. Integrating hydrogel design, biological response, and clinical outcomes within a continuous ML-guided framework could ultimately support the development of safer, more reliable, and clinically relevant drug-delivery systems.

Based on the literature reviewed, no hydrogel product designed or optimised with ML assistance has yet entered clinical use or reached the market. This lack of translation reflects limited and heterogeneous datasets, insufficient external and prospective validation, difficulty predicting in vivo biological responses from in vitro data, and unresolved safety, manufacturing, regulatory, and model-transparency requirements. Future breakthroughs will require standardised multi-institutional datasets, biologically informed and explainable models, prospective closed-loop experimental validation, and early integration of clinical endpoints, scalable manufacturing, and regulatory requirements into ML-guided optimisation.

## Figures and Tables

**Table 1:** Overview of major natural hydrogel biomaterials used in controlled drug delivery.

Material	Origin/Chemical Class	Crosslinking Mechanism	Key Properties	Representative Application	Ref.
Alginate	Anionic polysaccharide (brown algae)	Ionic (Ca^2+^, Sr^2+^)	Rapid, mild gelation; biocompatible; limited long-term network stability	Cell/protein encapsulation, wound dressings	[10]
Chitosan	Cationic polysaccharide (chitin deacetylation)	Polyelectrolyte complexation; chemical	Mucoadhesive, antimicrobial; pH-dependent solubility	Mucosal and wound delivery	[10]
Gelatin/GelMA	Methacrylated collagen derivative	Photocrosslinking (UV, via methacryloyl groups)	Cell-adhesive (RGD-retaining), MMP-degradable; modulus and pore size tunable via degree of substitution (~2–30 kPa; ~25–50 μm)	Localised antibiotic/anticancer delivery	[11]
Hyaluronic acid	Glycosaminoglycan	Chemical crosslinking; clinically used HA fillers are commonly crosslinked using BDDE (1,4-butanediol diglycidyl ether or DVS (divinly sulfone)	Viscoelastic, biocompatible, injectable and durable following crosslinking	Dermal fillers and soft-tissue augmentation	[12]

**Table 2:** Overview of major synthetic hydrogel biomaterials used in controlled drug delivery.

Material	Chemical Class	Crosslinking Mechanism	Key Properties	Representative Application	Ref.
Polyethylene glycol (PEG)	Synthetic polyether	Photopolymerisation (acrylate/thiol-ene)	Bio-inert, reproducible, tunable mesh size	Diffusion-controlled depots, PEGylated DDS	[14]
Polyvinyl alcohol (PVA)	Synthetic vinyl polymer	Physical (freeze–thaw crystallisation)	Tough, elastic, additive-free	Cartilage substitutes, wound films	[15]
PVA/PEG blend	Hydrogen-bonded composite	Physical, PEG-mediated stabilisation	Improved network stability, tunable swelling	Wound dressings, topical DDS	[16]
Poloxamer (Pluronic)	Synthetic PEO-PPO-PEO triblock copolymer	Thermoreversible physical gelation/self-assembly	Amphiphilic, thermoresponsive, grade-dependent properties	Drug delivery and biomedical formulations	[13]

**Table 3:** Overview of drug-release mechanisms in hydrogel-based delivery systems.

Release Mechanism	Driving Force	Rate-Controlling Factor	Advantages	Limitations	References
Diffusion controlled	Difference in drug concentration between the hydrogel and the surrounding medium	Drug diffusivity within the gel and the pore/mesh size of the hydrogel network	Relatively simple system in which drug release can often be estimated from diffusion behaviour	Initial burst release, less effective for large molecules	[5,13]
Swelling controlled	Water entering the hydrogel causes expansion of the polymer network	Rate of water uptake, polymer relaxation, and the extent of swelling	Drug release can be adjusted by modifying the swelling properties of the hydrogel	Sensitive to hydration and swelling behaviour	[4,8]
Degradation controlled	Breakdown of the polymer network by hydrolysis or enzymatic activity	Matrix degradation and erosion rate	Can provide drug release over longer periods and may be useful for larger therapeutic molecules	Difficult to accurately predict degradation in vivo	[10,12]

**Table 4:** Comparison of stimuli-responsive hydrogel systems for controlled drug delivery.

Stimulus	How the Hydrogel Responds	Influence on Drug Release	Main Consideration	References
Ph	Protonation/deprotonation of ionisable groups and swelling of network due to local pH change	Swelling and mesh size changes affect the drug diffusion	Response is dependent on amount and variation of pH changes at disease site	[49]
Glucose	Glucose reacts with PBA groups or is oxidised using GOx based system	Drug release is triggered by crosslinking changes, swelling or local pH.	H_2_O_2_ production by GOx systems suitable for glucose-regulated delivery	[51,58]
Redox/ROS	GSH can break reduction-sensitive disulphide bonds while ROS changes oxidation-sensitive groups	Loss/alteration of network structure permits therapeutic release	Response depends on local redox environment; ROS responsiveness relevant to oxidative injury after MI	[52]
Temperature	Polymer hydration and hydrophobic interactions that lead to network swelling or contraction	Changes in network architecture and mesh size affect drug diffusion and release	The transition temperature should be suitable for the intended physiological application	[55]
Light	Photocleavage or photothermal change induced by UV/visible or NIR light	Irradiation causes local changes in the matrix, followed by drug release	Provides external control, but penetration and phototoxicity depend on wavelength	[56]

**Table 5:** Comparative analysis of response mechanisms [74].

Response Type	Response Mechanism	Composition	Application	Advantage
pH Response	1. Solvation or contraction of ionisable groups (COOH, -NH_2_) of polymers 2. Acid-sensitive dynamic covalent bond degradation	Alginate, chitosan, carboxymethyl cellulose, oxidised dextran, bovine serum albumin (BSA)	pH-responsive degradation of ODex/BSA-Zn hydrogels via Schiff-base bonding	Dynamically adapts to the acidic environment of the wound for precise and targeted drug delivery; some hydrogels can actively regulate pH to promote macrophage polarisation
Enzyme Response	Use of protease-sensitive peptide chains, such as MMP-9, as crosslinking units to trigger hydrogel degradation or drug release after enzymatic digestion	Oxidised dextran, carboxymethyl chitosan, MMP-9-sensitive peptide chain	MMP-9 releases M2type macrophage exosomes in response to hydrogels and promotes M1→M2 polarisation to accelerate healing	Specific targeting of inflammatory sites, regulating abnormal enzyme activity, and promoting wound repair
ROS Response	ROS-sensitive chemical bond (thioketone, borate) breakage triggers degradation or drug release	Tannic acid (TA), polyvinyl alcohol (PVA), cerium dioxide (CeO_2_) nanoenzymes, phenylborate crosslinker	1. HAP- PVA/Reg3α hydrogel degrades and releases the drug in a high hydrogen peroxide (H_2_O_2_) environment 2. PBA-TA-PVA hydrogel cascade scavenges ROS	Dual function: responsive drug release + direct removal of oxidative stress to improve the wound microenvironment
Glucose Response	1. Glucose oxidase (GOx) catalyses the production of gluconic acid and H_2_O_2_ from glucose 2. Concanavalin A (ConA) binds competitively to glucose 3. Phenylboronic acid (PBA) reversibly binds to glucose	GOx, ConA, PBA	1. GOx hydrogel triggers insulin release via pH/ROS changes 2. PBA hydrogel binds glucose to regulate solubility	Real-time monitoring of blood glucose levels, on-demand drug release, can be integrated with other response mechanisms to build a multi-functional system

**Table 6:** Hydrogel local drug-delivery systems (HLDDS) for postsurgical management of tumours [78].

	Types	Advantages	Disadvantages
Non-in situ HLDDS	Implantable HLDDS	Certain rigidity; serve as postsurgical scaffold	Difficult to degrade; need a larger wound to implant; hard to completely conform postsurgical wounds and cavity
	Injectable HLDDS	Shear thinning; injectable; minimally invasive	Poor mechanical strength; high requirements on materials
In situ HLDDS	Thermo-sensitive in situ HLDDS	Easy to adjust, control and implement; convenient retrieval of thermo-sensitive materials	Natural biomaterials: poor plasticity; insufficient mechanical strength; synthetic material: suboptimal biocompatibility
	Ion-sensitive in situ HLDDS	Rapid response and excellent biocompatibility	Low mechanical strength; insufficient ions in tumour tissue fluid
	Photocuring in situ HLDDS	Remote control; being able to combine with PDT or PTT	Biosafety concerns like skin damage and chemical toxicity of photosensitisers
	Non-responsive in situ HLDDS	Suitable for chemical crosslinked hydrogels with good mechanical strength	Require a dual syringe or a dual-cartridge sprayer

**Table 7:** Natural, synthetic, and semi-synthetic hydrogels for 3D cell cultures [107].

Source of Hydrogels	Properties	Materials	Cell	Applications
Natural	Provide comparable viscoelasticity and fibrils to the ECM; having good biocompatibility; endogenous factors can support cellular activity	Collagen	Rat chondrocyte, hMSCs, rMSC, HUVECs/hASCs	Maintain the chondrocyte phenotype; facilitate chondrogenic differentiation of hBMSCs and rBMSCs; form stable EC networks; promote cell viability; promote growth of hMSCs.
		HA	hiPSC-NPCs, hiPSCs, rMSCs, human breast cancer MCF-7 cells, HepG2 cells, human dental pulp stem cells, hNS/PC, and hMSCs	Promote the neural differentiation of hiPSC-NPCs; cardiac differentiation of hiPSCs; osteogenic differentiation of human dental pulp stem cells; the adhesion and proliferation of HepG2 cells; cell spreading, fiber remodelling, and focal adhesion of hMSCs; maintain the stemness of rMSCs and induce the direct cartilage differentiation; enhance the tumorigenic capability of MCF-7 cells; increase the oligodendrocytes and neural differentiation of hNS/PC and support long-term cell viability.
		Fibrin	HUVECs/hMSCs, porcine cumulus–oocyte complexes (COCs), primary human chondrocytes, mHPSCs, and hiPSCs/HUVECs/human dermal fibroblast	Prevascular formation of HUVECs, improve cell viability and proliferation of hMSCs and enhance their osteogenic differentiation and bone mineral deposition; maintain the functional relationship between oocytes and follicular cells; induce the production of glycosaminoglycans and collagen type II of primary human chondrocytes; enhance the murine hematopoietic stem/progenitor cells (mHPSCs) expansion and differentiation; no effect viability and prevascular formation of encapsulated cells.
		Alginate	hESCs/hiPSCs, hiPSCs-derived neurons	Enhance the generation of retinal pigmented epithelium and neural retina of hESCs/hiPSCs; form complex neural networks.
Synthetic	Have the good mechanical strength to provide structural support for various cell types in 3D cell culture	PVA	mHSCs, mSCCs, human glioma cell lines LN299, U87MG and Gli36, human breast cancer Hs578T cells, and human pancreatic cancer cell lines Sui67 and Sui72	Enhance the expansion of murine hematopoietic stem cells (mHSCs); promote the meiotic and post-meiotic differentiation rate of mSCCs; form tumour spheroids.
		PEG	hiPSCs, mMSCs, chondrocyte, and hMSCs	Enhance the hematopoietic differentiation of hiPSCs; evaluate the behaviour of mMSCs and hMSCs at the specific condition; prolong the oxygen release of chondrocytes.
Semi-synthetic	Have a feature of ECM microenvironment and faster stress relaxation	HA–PEG	hiPS-HEPs and HUVECs	Enhance viability and functionality of hiPS-HEPs; promote the capillary-like sprouts formation of HUVECs spheroids.
		RGD–alginate–PEG	Fibroblasts and mMSCs	Increase the spread and proliferation of fibroblasts and the osteogenic differentiation of mMSCs.

**Table 8:** Relationship between key hydrogel characteristics and their representation as ML features.

Hydrogel Characteristic	ML Feature Representation	Relevance to Prediction	Ref.
Polymer composition	Polymer type, molecular weight, polymer concentration	Influences network structure, swelling, degradation, and drug-release behaviour	[102,105]
Crosslinking	Crosslinker concentration, crosslinking method/density and crosslinking conditions	Influences mesh size, molecular diffusion, mechanical stability, and drug retention	[108,109,110]
Swelling behaviour	Equilibrium swelling ratio, swelling percentage, or time-dependent swelling	Represents network expansion affecting mesh size and drug diffusion	[113,114]
Degradation behaviour	Mass loss (%), degradation rate or degradation at defined time points	Represents matrix persistence and degradation-controlled release	[115,116,117]
Mechanical properties	Compressive modulus, tensile strength, Young’s modulus, and viscoelastic parameters	Quantifies structural and mechanical performance	[119,120,121,122,123]
Drug–polymer interactions	Drug size, charge, solubility/affinity and polymer characteristics	Influences drug loading, retention, diffusion, and release	[1,10,18]
Drug size relative to mesh size	Molecular/hydrodynamic drug size relative to hydrogel mesh size	Represents steric restriction controlling drug diffusion	[8,16,22,23]

**Table 9:** Summary of machine-learning applications in hydrogel development stages.

Stage and Study	Hydrogel System and Dataset	ML Approach	Validation and Main Finding	Key Limitation	Ref.
Material selection: Liao et al.	Experimental library of 180 bioinspired adhesive hydrogels.	Nine regression models combined with sequence mining and de novo design.	A 90/10 split, 10-fold cross-validation and experimental testing were used; the selected hydrogel exceeded 1 MPa underwater adhesion.	The endpoint was adhesion rather than drug delivery, and no clinical validation was performed.	[135]
Formulation and inverse design: Cadamuro et al.	Gelatin-hyaluronic-acid hydrogels crosslinked through click chemistry; rheological data described storage and loss moduli.	A neural-network tool predicted formulation composition from target rheological properties.	Predicted formulations were prepared and rheologically tested, supporting inverse design within the studied composition space.	The model was restricted to one chemistry and did not evaluate release, biological efficacy, or external datasets.	[136]
Formulation optimisation: Seifermann et al.	High-throughput dataset of more than 900 photodegradable hydrogel formulations.	Gaussian-process modelling and Bayesian optimisation with iterative experimental selection.	A closed experimental loop identified formulations with improved photodegradation behaviour.	The endpoint was photodegradation, not drug release; transfer to other hydrogel families was not tested.	[137]
Formulation optimisation: Karaoglu et al.	Experimental GelMA library varying eosin Y, triethanolamine and N-vinylpyrrolidone; outputs were Young’s modulus and gelation time.	Artificial neural network for simultaneous prediction of stiffness and gelation time.	Predictions were compared with experiments, and cell-survival testing defined nontoxic component ranges.	Single GelMA-photoinitiator system; no independent external or in vivo validation.	[138]
Wound monitoring and repair: Deng et al.	A pH-responsive GelMA-based hydrogel dressing combined with smartphone images of wound pH.	ML-assisted image analysis for estimating wound pH.	The hydrogel demonstrated antibacterial, anti-inflammatory, antioxidant and angiogenic activity and improved diabetic wound healing in mice.	ML estimated wound pH rather than directly predicting wound-healing outcomes. No clinical validation was performed.	[139]
Anti-inflammatory biological performance: Han et al.	Hydrogel scaffold data linking storage modulus, loss modulus, swelling ratio, porosity, pore size, and roughness with M2 macrophage polarisation.	Interpretable ML, feature ranking and sequential optimisation of physicochemical properties.	Combinations of hydrogel properties were identified to promote anti-inflammatory M2 macrophage polarisation.	The biological endpoint was limited to a cell-level immune response, without patient-level validation.	[140]
Rheological characterisation: Mohammad et al.	Laboratory formulation and rheology data for 3D-printed polyacrylamide hydrogels.	MLP predicted storage and loss moduli; VAE and CVAE generated candidate compositions.	Grid search with 10-fold cross-validation produced R^2^ = 0.89; generated data were assessed statistically and with anomaly detection.	One material system; synthetic-data agreement does not replace independent experimental validation.	[141]
Mechanical characterisation: Xu et al.	Literature-derived dataset of 350 PVA hydrogel records.	XGBoost with Optuna optimisation and SHAP interpretation for tensile-strain prediction.	Reported R^2^ values were 0.964 for training and 0.801 for testing.	Literature protocols were heterogeneous, and prospective experimental validation was not reported.	[142]
Antitumour biological performance: Li et al.	Injectable hydrogel for the co-delivery of CAR-T cells and the mitophagy agonist BC1618 for triple-negative breast cancer.	AI-based screening of mitophagy-active compounds followed by experimental hydrogel development.	The system improved CAR-T-cell activity and antitumour efficacy in vitro and in a mouse tumour model.	AI selected the therapeutic compound rather than optimising the hydrogel formulation. No clinical validation was performed.	[143]
Drug-release optimisation: Cheong et al.	Automated seed library of 120 alginate hydrogels containing bovine serum albumin.	Gaussian-process regression with active learning to optimise cumulative release profiles.	Model-recommended formulations were tested prospectively through iterative automated experiments.	One polymer and a model protein were used; in vivo and clinical validation were not performed.	[144]
Biological and preclinical validation: Li et al.	Nine PubChem and DeepChem bioactivity datasets used to screen nucleoside hydrogel candidates.	Decision tree, logistic regression, random forest and XGBoost combined with multi-criteria screening.	GMP and dGMP hydrogels demonstrated biocompatibility, antibacterial and anti-inflammatory activity and tissue regeneration in mouse periodontitis models.	Validation remained preclinical, with no independent human dataset or patient-level outcome assessment.	[145]

**Table 10:** Translational barriers and proposed strategies for ML-guided hydrogel development.

Translational Barrier	Potential Effect	Proposed Strategy	References
Limited dataset size	Model overfitting and poor generalisability	Develop multi-institutional databases and include successful and unsuccessful formulations	[151,152]
Heterogeneous experimental and reporting methods	Limited comparability and difficulty combining datasets	Standardise formulation descriptors, measurement units and testing protocols	[151,153]
Gap between in vitro and in vivo performance	Reduced accuracy when predicting biological and clinical outcomes	Incorporate physiologically relevant models, animal data and independent clinical validation	[154,155]
Safety, degradation and immunogenicity concerns	Selection of formulations that perform well experimentally but remain clinically unsuitable	Include toxicity, degradation and immune response as constraints in multi-objective optimisation	[155,156]
Manufacturing and regulatory requirements	Poor reproducibility and difficulty obtaining regulatory approval	Integrate quality-by-design principles, manufacturing variability and transparent model documentation	[156,157]

**Table 11:** Minimum reporting and validation requirements for ML-guided hydrogel studies.

Reporting or Validation Area	Minimum Information Required	Purpose	References
Dataset description	Sample size, data sources, inclusion criteria, missing values, and distribution of outcomes	Allows evaluation of dataset quality and possible bias	[151,152]
Formulation and synthesis information	Polymer type, molecular weight, concentrations, crosslinking method, and processing conditions	Supports reproducibility and comparison across studies	[151,153]
Outcome measurement	Testing protocols, measurement units, environmental conditions, and assessment time points	Ensures that model outcomes are clearly and consistently defined	[153,154]
Data splitting and leakage control	Separation according to experimental batch, study, material family, or formulation	Prevents closely related observations from appearing in both training and testing datasets	[152,155]
Model performance and uncertainty	Multiple performance metrics, calibration, confidence intervals, and uncertainty estimates	Provides a more reliable assessment of predictive performance	[155,157]
External and prospective validation	Testing using independent datasets and experimental evaluation of predicted formulations	Demonstrates generalisability and practical utility	[154,156]
Reproducibility	Model parameters, software, code, and access to appropriately anonymised data	Enables independent verification and future model development	[152,157]

## Data Availability

No new data were created or analyzed in this study. Data sharing is not applicable to this article.
